# Code-driven planning in grid worlds with large language models

**DOI:** 10.1098/rsos.260255

**Published:** 2026-10-21

**Authors:** Ashwath Vaithinathan Aravindan, Zhisheng Tang, Mayank Kejriwal

**Affiliations:** ^1^ University of Southern California, Los Angeles, CA, USA; ^2^ Information Sciences Institute, University of Southern California, Los Angeles, CA, USA

**Keywords:** code-driven planning, large language models, spatial common sense reasoning, MiniGrid

## Abstract

We propose an iterative programmatic planning (IPP) framework that solves grid-based tasks by using large language models (LLMs) to synthesize interpretable, executable programs that act as agent policies mapping environment states to action sequences, rather than relying on traditional search or reinforcement learning. Alongside prompting strategies such as direct code generation, pseudocode conditioned refinement and curriculum-based prompting, IPP centres on an iterative refinement mechanism that updates code from coarse, task-level feedback. We evaluate our approach using six leading LLMs and two challenging grid-based benchmarks (grid-based reasoning for assessing spatial planning (GRASP) and MiniGrid). Our IPP framework demonstrates improvements over direct code generation for five of the six models, with net energy gains ranging from a few per cent to roughly threefold and, for Gemini-2.5-Pro, a sign flip from a negative baseline to a strongly positive score, establishes a new state-of-the-art result for GRASP and significantly outperforms direct elicitation from GPT-o3-mini (by 43% on the hardest MiniGrid task to 116% on GRASP). While code generation has a higher initial prompting cost compared to direct solution elicitation ($0.08 per task versus $0.002 per instance for GPT-o3-mini), the code can be reused for any number of instances, making the amortized cost significantly lower (by 400 times on GPT-o3-mini across the complete GRASP benchmark). Beyond performance gains, IPP produces reusable, human-readable policies that support transparent inspection and modification, underscoring the advantages of planning as program synthesis over per-instance inference. The code and results can be found at http://github.com/Mystic-Slice/LLM-Planning-CodeGen.

## 1. Introduction

Grid-based environments are a core abstraction for planning tasks in robotics [1–3], embodied AI [4] and reinforcement learning (RL) [5–8]. These environments offer a formal, discrete setting to study core aspects of intelligent behaviour, such as planning [9,10], exploration [11] and generalization [12]. Their simplicity enables systematic evaluation across diverse approaches: from classical search [13] and RL [14,15] to neurosymbolic [16] and language-based methods [17]. They are commonly used as a simplified abstraction of domains such as robotics and navigation, capturing spatial and symbolic reasoning while stripping away perception noise and sensor complexity. We treat them not as a stand-in for real-world robotics, which involves continuous, noisy and only partially observable dynamics, but as a controlled setting in which decision-making and planning can be studied and compared systematically across methods.

Meanwhile, large language models (LLMs) have recently shown strong performance on a variety of reasoning tasks, including question answering [18,19], tool use [20,21] and code generation [22,23]. However, LLMs have struggled with planning [24], which requires state tracking, goal-directed search and consistent multi-step decision-making, all of which LLMs do not naturally exhibit. At the same time, LLMs excel at generating interpretable code [23], which motivates a shift in perspective: instead of prompting LLMs to produce action plans directly, we treat planning as code generation. LLMs can generate executable programs that act as action policies. This capability aligns *planning with programming*, since both require symbolic reasoning over structured inputs and constraints. Additionally, such an approach offers several advantages: generated programs are interpretable, reusable across task instances and cheap to execute.

Compared to RL, which relies on trial-and-error interaction and costly gradient-based optimization, a code-driven approach offers a lightweight and interpretable alternative that requires no task-specific training. While RL excels in grid-based domains, it often suffers from poor generalization [25,26] and produces opaque policies [8,27]. By contrast, a code-driven approach replaces policy learning with LLM-based program synthesis, enabling the generation of symbolic and reusable policies that are efficient and transparent and aligning with a broader shift towards grounding LLMs in executable environments [28–30] where code serves as a verifiable and editable interface between high-level reasoning and low-level control. For the class of problems we consider, namely planning with deterministic, fully observable dynamics that are known in advance, generated code can serve as a lightweight and practical alternative to RL for building generalizable planning agents. Settings with stochastic transitions, partial observability or dynamics that must be learned through interaction still call for RL or other methods that explicitly reason about uncertainty.

In this work, we propose **iterative programmatic planning (IPP)**, a code-driven framework that uses LLMs to generate Python programs as action policies. IPP incorporates an **iterative refinement (IR)** loop, where LLM-generated programs are executed, evaluated and revised based on feedback to correct errors and improve task performance. Iterative self-refinement, in which a model revises its own outputs in response to feedback, is by now a well-established idea in the LLM literature [31,32]. What is new in IPP is how this idea is instantiated for planning: the refined artefact is a single, reusable policy program rather than a natural language answer or chain of thought (CoT), and refinement is driven solely by a coarse, task-level signal such as reward rather than fine-grained or per-step supervision. Studying this setting across six models and two benchmarks, we find that such coarse feedback is often sufficient to iteratively synthesize policies that match or exceed strong baselines. This also sets IPP apart from prior code-as-policy systems such as Voyager [33], which incrementally grows a library of skills through environment interaction, and WorldCoder [34], which synthesizes a code-based model of the environment's dynamics; IPP maintains no skill library and learns no world model, treating the policy program itself as the object to be iteratively improved. Moreover, whereas Voyager keeps an LLM in the loop throughout its lifetime to propose tasks and author skills, IPP invokes an LLM only during a bounded refinement phase, after which the synthesized program is executed on any number of task instances without further LLM calls.

Figure 1 illustrates one example of performance improvement enabled by IR. We evaluate IPP on two grid-based benchmarks, namely grid-based reasoning for assessing spatial planning (GRASP) [35] and MiniGrid [7], across six state-of-the-art LLMs, and show that it produces robust and generalizable *code-based policies*. Compared to directly prompting for action sequence and other baselines, IPP offers better performance, greater transparency and lower computational cost. In summary, this work makes the following contributions:

— We propose IPP, a novel framework that treats planning as code generation. Instead of producing action sequences directly, LLMs synthesize interpretable Python programs as reusable policies for grid-based environments. IPP introduces an *IR* process that improves these programs based on task-specific feedback. This approach offers transparency, efficiency and generalization without requiring task-specific training.

**Figure 1.**
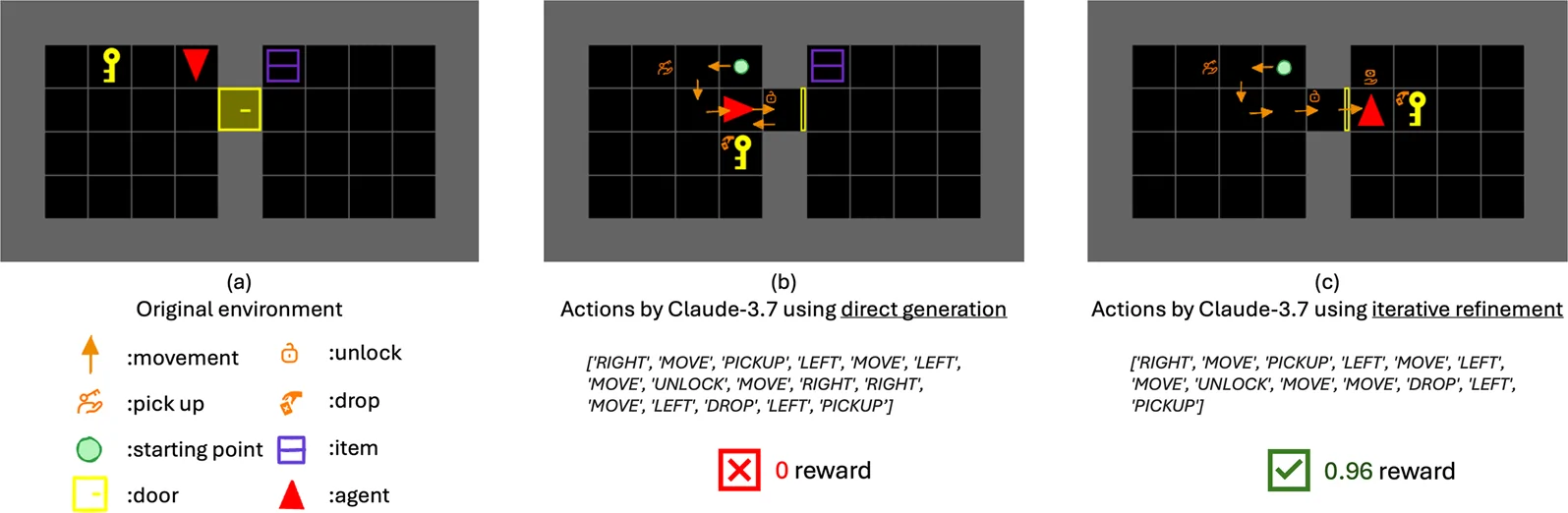
Example of the *Unlock-Pickup* task from the MiniGrid benchmark. The agent (red triangle) must retrieve a box (purple item) located in a separate room behind a locked door (yellow). Directions in the action sequence indicate the direction the agent turns to face before moving. Panel (a) shows the original environment set-up. Panel (b) shows actions generated by Claude-Sonnet-3.7 using *direct generation* (DG), which fails and yields a reward of 0. Panel (c) shows *IR*, which successfully completes the task with a reward of 0.96.
— We evaluate IPP on two benchmarks: GRASP and MiniGrid, across six leading LLMs. IPP improves the average net energy over direct code generation on GRASP for five of the six models (for example, a threefold increase for GPT-o1, from 0.94 to 2.82, and turning Gemini-2.5-Pro's negative baseline into a strongly positive score, from −0.52 to 3.22) and raises GPT-o3-mini's reward on the hardest MiniGrid task, Unlock-Pickup, from 0.00 to 0.78. Compared to non-code-based CoT and two-step CoT prompting, IPP attains equal or better performance at significantly lower cost, since its synthesized policies generalize without requiring per-instance inference.
— We introduce two structured prompting strategies: *pseudocode extension*, where LLMs turn a high-level algorithmic sketch into executable code, and *step-by-step curriculum prompting*, where the complexity of the task is incrementally added. In the GRASP benchmark, pseudocode extension improves energy collection for Claude-Sonnet-3.7 from 0.89 (Greedy) to 0.98 (+10%) and for GPT-o3-mini to 1.36 (+52%). Using step-by-step curriculum prompting, GPT-o3-mini achieves steady gains—from 0.44 to 1.73 (+293%) at final complexity—while Claude-Sonnet-3.7 improves from 0.12 to 1.42 (+1083%) from Step 0 to Step 2 before levelling off, demonstrating the benefit of curriculum-based synthesis.

## 2. Preliminaries

### 2.1. Code as policy

LLMs have demonstrated strong capabilities in generating executable and interpretable code across a variety of domains. Hence, in RL settings, they have been explored for use as policy synthesizers [36,37]. The code itself acts as a policy, as a medium to define conditional logic, loops and decision structures that generalize beyond a single instance.

### 2.2. In-context learning

LLMs exhibit an ability to condition their output on examples, task descriptions or structured prompts presented within its input context, known as in-context learning (ICL) [38]. More specifically, in our work, we use a form of ICL known as few-shot prompting [39], by providing a small number of input–output examples within the prompt to guide the model's behaviour on new, unseen instances. Although it is generally used to communicate task structure, format and reasoning patterns, we leverage this capability for policy refinement in the proposed IPP framework.

### 2.3. Planning problems

We consider planning problems that can be represented as discrete, grid-based environments. We assume that each environment is fully observable and composed of cells, and each cell is associated with attributes such as obstacles, items and goal-relevant entities. Agents must navigate these grids and execute a task—for example, item collection or goal reaching—by performing valid action sequences that satisfy task-specific constraints. These environments are common in domains, such as embodied AI, robotics and RL [2,4,7].

Formally, each planning problem is represented as a tuple $P=(S,A,T,s_{0},G)$, where S is the state space; each s∈S denotes a configuration of the grid environment, including the agent's position and attributes (e.g. energy and inventory), as well as locations of objects and obstacles. A is the finite set of discrete actions (e.g. move, pick up, unlock and drop) that the agent is allowed. T:S×A→S is the predefined transition function. We assume that the transition function is deterministic and fully defined, so the effects of each action in a given state are predictable and known. $s_{0}\in S$ is the initial state. We assume that this is fully observable, meaning that the agent has access to the complete initial state of the environment. Finally, G⊆S is the set of goal states.

A valid plan is then defined as a sequence of actions $\pi =[a_{0},a_{1},\ldots ,a_{T}]$ such that the resulting state after executing π from $s_{0}$ falls within the goal region: $T(T(\ldots T(s_{0},a_{0}),a_{1}),\ldots ,a_{T})\in G$. The agent's task is to generate a sequence of actions $\pi =[a_{0},a_{1},\ldots ,a_{T}]$ that transforms the initial state $s_{0}\in S$ into a goal state $s_{T}\in G$ under the transition function T. This requires reasoning about the structure of the environment, anticipating the effects of actions via T(s,a) and selecting actions from A that satisfy task-specific constraints while getting closer towards the objective.

## 3. Approach

To solve such planning problems, we propose an IPP framework that uses LLMs to synthesize interpretable and executable programs that serve as action policies. In this framework, the LLM generates programs that map environment observations to sequences of allowable actions, effectively functioning as planning agents. We first decompose the problem into three key components: (i) *direct program synthesis*, where the LLM is prompted to generate complete, executable programs in a general purpose interpretable language (e.g. Python) from natural language task descriptions; (ii) *pseudocode conditioned refinement*, where the model is seeded with a high-level algorithmic sketch (e.g. greedy search) and tasked with completing or adapting it to the task environment; and (iii) *curriculum-based prompting*, where the model is guided through a sequence of incrementally complex subproblems to facilitate generalization to harder tasks.

We combine these components into a unified prompting-based approach^1^ suitable for zero- and few-shot planning through code generation. However, to further *optimize* the program generated by the model, we also incorporate an IR process whereby the LLM first generates an initial candidate program, which is then executed and evaluated on a set of task instances. Based on some performance metric (such as reward), the model is prompted to revise the code. Iteration continues until performance converges or improves no further. Through refinement, we show that the LLM learns to correct logical errors, incorporate missing constraints and restructure control flow.

### 3.1. Code-driven planning with iterative refinement

Rather than relying on traditional search algorithms or RL to solve P, code-as-plan frameworks use an LLM to generate an interpretable program f that, when executed against a specific environment instance, produces a sequence of actions $\pi =[a_{0},a_{1},\ldots ,a_{T}]$ transitioning the agent from the initial state $s_{0}$ to a goal state $s_{T}\in G$. This program acts as a reusable and interpretable policy that maps environment observations to action sequences.

This approach contrasts with classical RL methods, which typically learn a policy π:S→A through repeated interaction with the environment and optimization of cumulative rewards, a process that is time consuming and computationally intensive. At the other extreme, an LLM could be prompted to directly produce a plan given a grid-based planning problem. Experimentally, this approach is found to yield significantly low-quality solutions, even for leading LLMs (§5; see also figure 1). Code-driven planning offers a compromise by forcing the LLM to provide an explicit policy, using only a few prompts, encoded as an interpretable program. An auxiliary advantage of such a policy is that its logic is observable and interpretable.

*Direct generation (DG)*. Our baseline approach, *DG*, prompts the LLM to directly synthesize a complete and executable program f given only a natural language task description and environment specifications. This approach assumes that the model can generate code that considers both a high-level strategy and low-level execution details in a single pass. While fast and straightforward, this approach may overlook edge cases or misapply rules. Nonetheless, it establishes a strong starting point for comparing more advanced techniques. Decomposing a complex task into a planning phase followed by an execution phase has been shown to improve reasoning quality in LLMs [40]. Accordingly, as Step 1, we prompt the model to generate only the algorithm's pseudocode. After receiving this intermediate output, we proceed to Step 2, in which the model generates the final code according to a fixed function API. This two-stage procedure not only improves code correctness but also provides greater interpretability, as the intermediate planning step reveals the model's reasoning before code synthesis.

*Pseudocode extension*. To address some of the flaws of DG, we introduce a pseudocode-based prompting strategy. Here, the LLM is provided with a high-level baseline method (e.g. greedy or rule-based logic in pseudocode) and tasked with extending and refining it into an executable program f. This approach narrows the LLM's search space, allowing the model to use *symbolic priors* while preserving flexibility. A disadvantage of this approach is that it requires the pseudocode prior to be provided in the first place. For complex grids, providing such a prior may be cumbersome or even infeasible.

*Step-by-step*. The *step-by-step* approach introduces a curriculum style procedure, in which the model is prompted to solve increasingly complex versions of the task, gradually building up to the full problem. This curriculum design follows the same least-to-most prompting principle of decomposing a hard problem into a sequence of simpler subproblems [40]. The step-by-step approach is purely prompting based: at each step, it is asked to update its code to accommodate one additional parameter or constraint, building up to the full task. Beginning with simplified environments (e.g. no obstacles, no step cost), the model produces initial code $f^{\left(0\right)}$ that works under minimal assumptions. At each subsequent stage i, the model is given an updated problem with more complexity, such as limited energy, obstacles or carry constraints, and is required to revise and extend the previous program $f^{\left(i-1\right)}$ into a new program $f^{\left(i\right)}$ that can solve the current problem. This formulation mimics human incremental reasoning and, in theory, should help the LLM produce a strategy that it might fail to produce through (for example) one pass DG.

*Iterative refinement*. To improve the correctness and generality of the generated program f, we propose an *IR* strategy, whereby an LLM iteratively improves its generated code through alternating steps of evaluation and feedback-driven refinement (algorithm 1; see also the flow chart in figure 2). Starting from an initial candidate program $f^{\left(0\right)}$ (obtained via DG), we evaluate its performance on a training set of planning problems P and measure its performance via a predefined task-specific metric $J^{\left(i\right)}$ for each instance.

**Figure 2.**
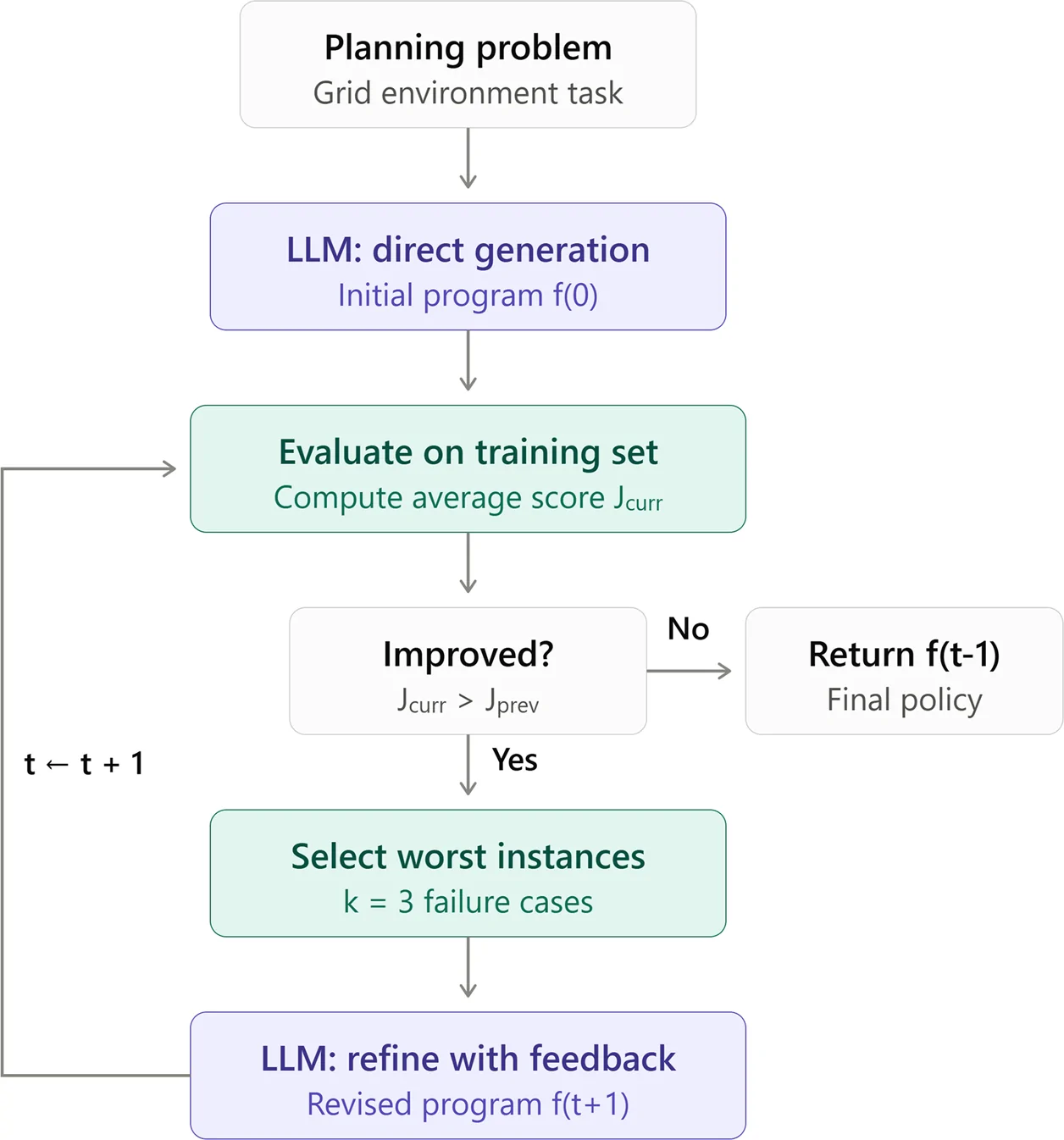
Flow chart of the IR process. Starting from an initial program $f^{\left(0\right)}$ produced by DG, IR repeatedly evaluates the current program on the training set and computes its average score $J_{\mathrm{curr}}$. If the score improves over the previous iteration, the k=3 worst-performing instances are selected as feedback, and the LLM is prompted to produce a revised program $f^{\left(t+1\right)}$; otherwise, the loop terminates and the previous program $f^{\left(t-1\right)}$, i.e. the best-performing iterate, is returned as the final policy. This mirrors the steps of algorithm 1.

**Algorithm 1. Iterative refinement.**

**Figure:**
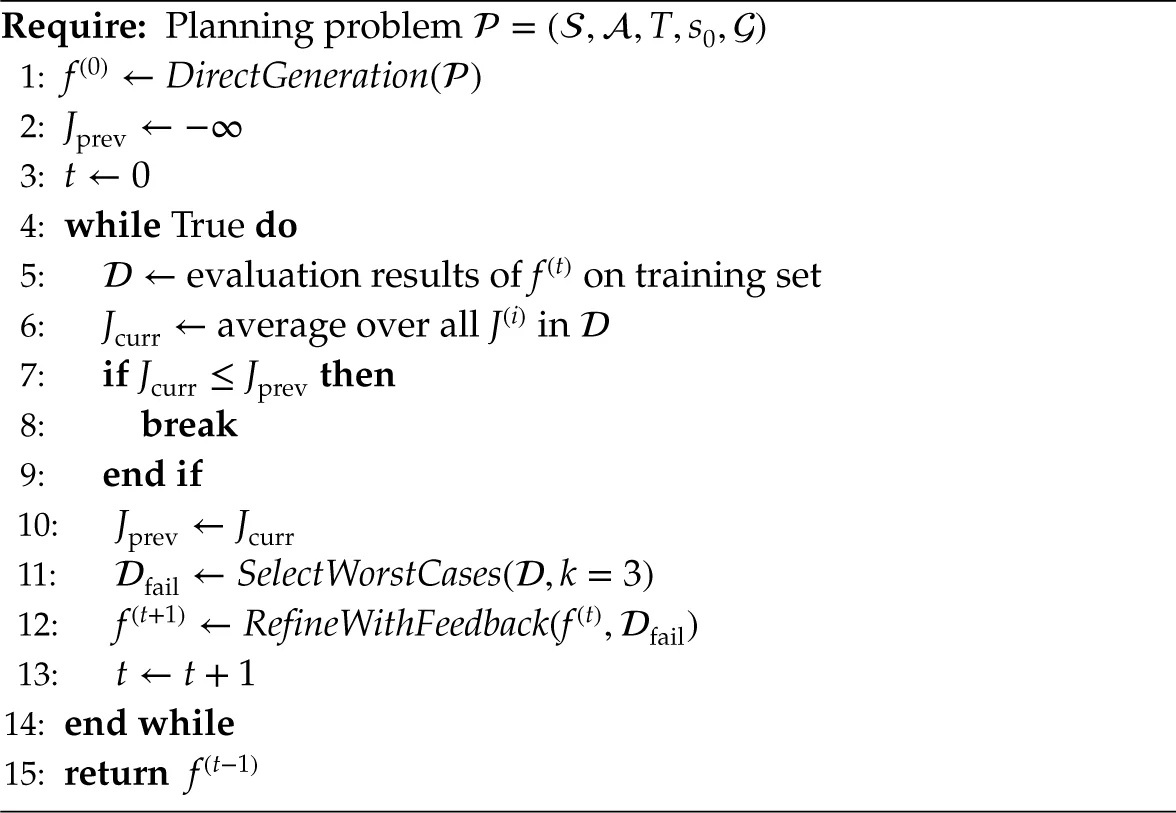

The aggregate performance $J_{\text{curr}}=\mathrm{Aggregate}(J^{\left(i\right)})$ is then compared to the previous iteration's performance $J_{\text{prev}}$. If no improvement is observed, we assume convergence and terminate the loop, returning the previous program $f^{\left(t-1\right)}$, i.e. the best-performing iterate. Otherwise, we identify the k worst-performing instances (i.e. those with the lowest $J^{\left(i\right)}$ values) as $D_{\text{fail}}$ and prompt the LLM to refine the current program $f^{\left(t\right)}$ based on this feedback. The revised program $f^{\left(t+1\right)}$ is expected to address the identified issues while preserving correct behaviour elsewhere.

From an optimization perspective, IR can be viewed as a discrete, non-gradient-based search procedure over the space of executable programs. Each refinement step attempts to improve a program $f^{\left(t\right)}$ by minimizing an implicit loss function, which is captured via task-specific evaluation metrics, over a finite dataset of grid instances. The feedback-driven update acts as a heuristic gradient, guiding the LLM to generate a new program $f^{\left(t+1\right)}$ that corrects previous failures while maintaining performance on successful instances. While analytically not an optimizer, IR approximates iterative function minimization in a symbolic space, guided by performance signals rather than gradients. In contrast to the step-by-step strategy, IR gives the model all of the problem's constraints from the outset and improves the code based specifically on the instances where it fails.

## 4. Experimental set-up

We evaluate our proposed framework on two benchmarks: the GRASP dataset [35] and three tasks from MiniGrid [7]. They are chosen for their structured two-dimensional grid environments, which can be easily converted into LLM prompts (see supplementary material, appendix C).

### 4.1. Evaluation benchmarks

#### 4.1.1. GRASP

The GRASP benchmark [35] is a large-scale, synthetic dataset designed to evaluate common sense spatial reasoning in autonomous agents, with a particular emphasis on planning and action selection rather than mere interpretation of spatial descriptions. Each GRASP environment is an 11×11 two-dimensional grid comprising cells that may be empty, contain a single unit of energy or be blocked by an obstacle. The agent is given full knowledge of the grid layout at the start of an episode and is allowed a maximum of 20 steps to act. At each step, the agent may execute movement-related actions to traverse the grid and resource-related actions to collect (TAKE) or deposit (DROP) energy. The objective is to maximize the total amount of energy successfully collected and dropped back at the starting cell within the step limit, reflecting practical scenarios in which agents must trade off exploration, exploitation and return costs. A sample GRASP grid instance is shown in figure 3.

**Figure 3.**
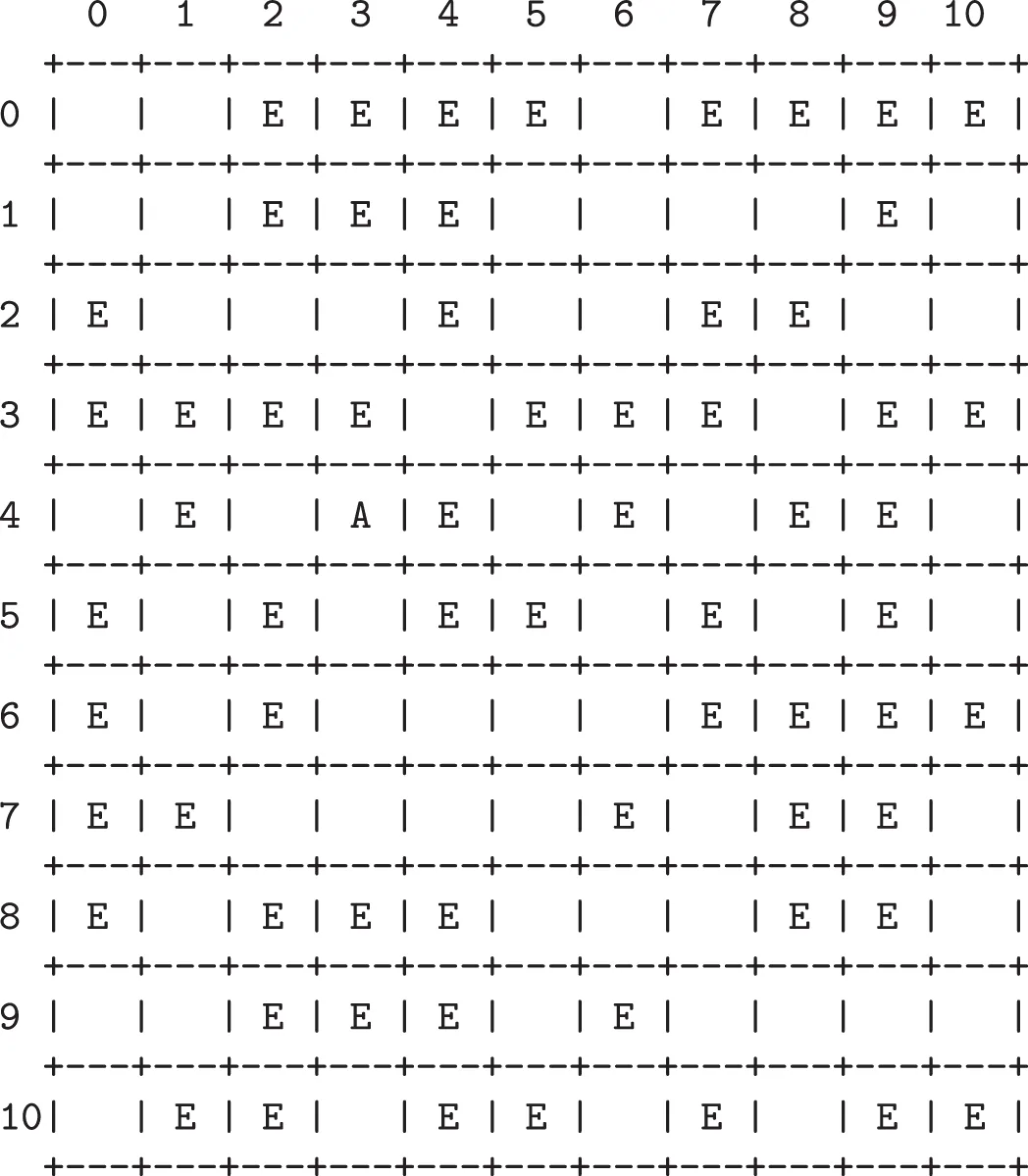
A sample GRASP grid instance. Each cell of the 11×11 grid is either empty, contains a single unit of energy (E) or is blocked by an obstacle (O); the agent (A) begins from a designated starting cell. The agent must plan a route that collects energy tokens and returns them to the starting cell within the step limit while minimizing movement cost.

The benchmark systematically varies grid configurations along three orthogonal dimensions: energy distribution, obstacle configuration and agent starting position. Five distinct energy distribution patterns are used to induce diverse spatial structures. In the random distribution, each cell independently contains energy with a probability sampled uniformly between 0.3 and 0.7. Vertically skewed and horizontally skewed distributions introduce asymmetry by assigning higher energy probabilities to either one half of the grid along the vertical or horizontal axis, with complementary probabilities applied to the remaining half. The cluster distribution concentrates energy into several randomly placed 3×3 clusters, encouraging region-based reasoning. The spiral distribution generates a continuous, spiral-shaped trail of energy radiating outward from the grid centre, testing the agent's ability to recognize and follow extended spatial patterns. Each grid instance uses exactly one of these five distributions. Obstacle configurations further modulate navigational complexity. In grids with obstacles, each cell can become an obstacle, rendering it impassable and unusable for energy collection.

Moreover, there are additional configurable parameters that add to the overall complexity of route optimization:

— Cost per step: energy expenditure for every action taken by the agent that encourages minimalist solutions.
— Carry limit: an upper limit on the number of energy tokens allowed to be carried by the agent simultaneously.
— Direction of movement: the agent movement can be limited to the four cardinal directions (UP, DOWN, LEFT and RIGHT) or can be allowed to move in also the four diagonal directions (UPRIGHT, UPLEFT, DOWNRIGHT and DOWNLEFT) increasing navigational flexibility.

By combining varied spatial layouts, explicit resource management objectives and multiple interacting constraints within a fully observable environment that yield up to 16 000 unique task instances, the benchmark probes whether an agent can transform spatial knowledge into coherent plans that balance efficiency, feasibility and goal satisfaction.

We evaluate performance on GRASP using the *average net energy*. For a single instance, the net energy is defined as

(4.1) $$E_{\mathrm{net}}=E_{\mathrm{collected}}-\left(N_{\mathrm{actions}}\times c_{\mathrm{step}}\right),$$

where $E_{\mathrm{collected}}$ is the total energy the agent successfully picks up and returns to its starting cell, $N_{\mathrm{actions}}$ is the number of actions it takes and $c_{\mathrm{step}}$ is the cost per step. The metric reported for each configuration is $E_{\mathrm{net}}$ averaged over all of its instances. Higher net energy indicates better performance. Because the action cost is subtracted, the metric can be negative when an agent expends more energy moving than it manages to collect and return.

#### 4.1.2. MiniGrid

MiniGrid [7] is a collection of lightweight, configurable two-dimensional discrete grid-world RL environments designed to facilitate reproducible and scalable evaluation of agents on sparse, goal-oriented tasks. Each environment comprises a grid world populated with walls and interactive objects (e.g. doors, keys and boxes) and parametrizes the difficulty by grid size and object layout. The default action space for IPP experiments is limited to a discrete set of six actions: LEFT, RIGHT, MOVE, PICKUP, DROP and UNLOCK. Episodes terminate upon task completion or when a specified maximum step count is reached, and rewards are typically sparse (e.g. near-binary with a small shaping component) to reflect successful task completion. For experiments evaluating IPP on MiniGrid, three tasks were chosen with increasing complexity.

— **Unlock**. In the Unlock task, the objective is to open a locked door that separates the agent from an exit region. The task requires the agent to locate and retrieve a key and then use an unlock action to unlock the door. A sample instance is shown in figure 4a.

**Figure 4.**
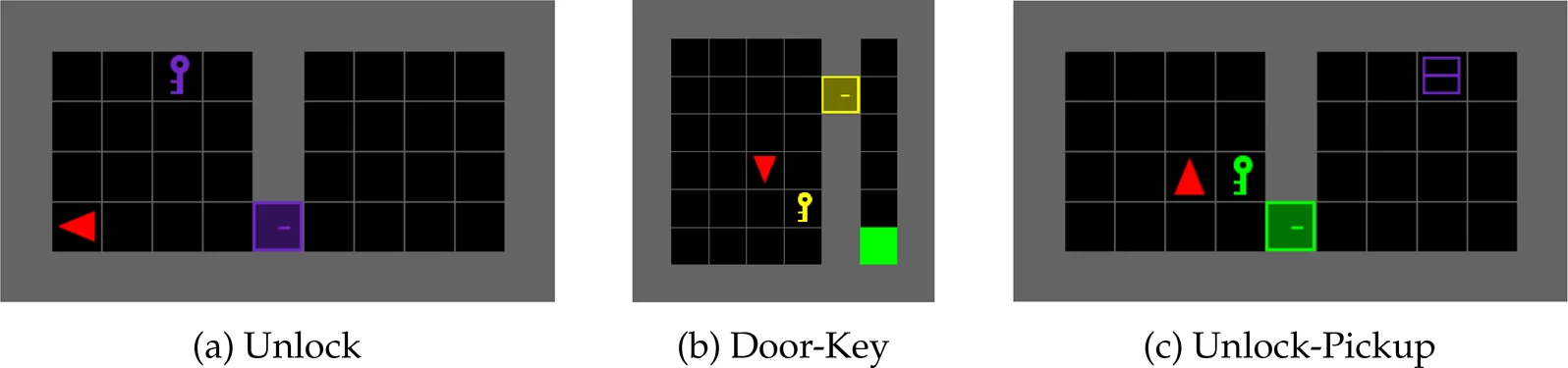
Sample instances of the three MiniGrid tasks used to evaluate IPP, shown in order of increasing complexity. The red triangle is the agent and grey cells are walls. (a) Unlock: the agent must retrieve the key and use it to unlock the door. (b) Door-Key: after unlocking the door, the agent must additionally navigate to the green goal square. (c) Unlock-Pickup: the agent must pass through the locked door to reach and pick up the target box in the adjacent room.
— **Door-Key**. The Door-Key environment generalizes the unlocking challenge by embedding it within a larger navigation task: the agent must first pick up a key, use it to unlock a locked door and then navigate to a designated goal location (a green goal square). A sample instance is shown in figure 4b.
— **Unlock-Pickup**. In the Unlock-Pickup task, the agent must traverse through a locked door to reach and pick up a target box located in a separate room. A sample instance is shown in figure 4c.

Each task adds on to the previous one thereby increasing the problem complexity. For each task, we generate 1000 instances and measure the average reward.

### 4.2. Prompting strategies and baselines

We evaluate the IPP framework using four prompting strategies, namely, *DG, pseudocode extension, step-by-step* and *IR* which were explained in detail in §3.1. Of these, pseudocode extension and step-by-step are applied only to the GRASP benchmark, whereas DG and IR are evaluated across both benchmarks. This scoping follows directly from the structure of the two benchmarks. GRASP exposes a rich set of independently configurable parameters—cost per step, carry limits, obstacle density and the set of permitted movement directions—that together span a wide and continuously tunable range of task complexity. This structure is what makes pseudocode extension and step-by-step meaningful: it affords a genuinely high-level algorithmic sketch that the model must adapt to many parameter regimes, and it allows a graded curriculum to be built by introducing these constraints one at a time. The MiniGrid tasks, by contrast, are defined by a small, fixed set of interaction rules rather than tunable difficulty axes. Because the intended behaviour on these tasks is already captured by a compact handwritten greedy routine, providing an equivalent pseudocode prior would largely amount to supplying the solution itself, leaving little room for the model to extend or improve upon it. For the same reason, the tasks cannot be decomposed into a sequence of progressively harder subproblems as the step-by-step curriculum requires. We therefore reserve these two structured strategies for GRASP, where their underlying assumptions hold. Additionally, we consider four non-code-based prompting strategies for plan generation. The simplest of them is *naive prompting*. In this setting, the model is instructed to output only a valid sequence of actions that completes the task, with no accompanying explanation, intermediate reasoning or auxiliary text. The output format is explicitly constrained to a list of actions. This strategy tests the model's ability to implicitly solve the planning problem in a single forward pass, without any external scaffolding or decomposition. While computationally efficient, naive prompting provides no mechanism for structured reasoning or error mitigation and serves as a strong baseline for evaluating the added value of more elaborate prompting schemes. To encourage more explicit reasoning, we adopt *CoT* prompting [41]. Under this strategy, the model is prompted to reason through the task in natural language before producing the final action plan.

We further introduce a *two-step CoT* variant designed to reduce the cognitive burden of generating long plans in a single pass. In this variant, planning is explicitly divided into two stages. In the first stage, the model generates a partial action sequence up to a predefined midpoint in the task horizon. The environment is then deterministically updated according to this partial plan, and the model is prompted a second time to complete the remaining portion of the plan based on the updated state. This staged decomposition enables more localized and focused reasoning at each step, reducing error propagation and allowing the model to condition later decisions on anticipated intermediate outcomes.

In addition to language-based prompting strategies, we evaluate two simple programmatic baselines that do not rely on learned reasoning or language modelling. These baselines provide reference points for interpreting task difficulty and contextualizing the performance of IPP.

The *random* baseline uniformly samples from the set of valid actions at each step. While this agent is not expected to succeed except by chance, it establishes a clear lower bound on performance and helps characterize the sparsity and structure of the action space.

The *greedy* baseline follows a hand-coded, myopic policy that selects actions based on immediate heuristic utility or apparent progress towards the goal. Although computationally efficient and deterministic, this policy does not account for long-term dependencies, delayed rewards or irreversible mistakes and therefore may fail on tasks requiring foresight or backtracking. As such, it provides a useful contrast to both natural language planning and code-based approaches.

### 4.3. Large language models evaluated

We evaluate our IPP framework using six LLMs, including both general-purpose LLMs and code-specialized ones. Specifically, we use the following: GPT-4o [42], GPT-o1 [43], GPT-o3-mini [44], Claude-Sonnet-3.7 [45], Gemini 2.5 Pro [46] and DeepSeek-R1 [47]. All models are accessed through the OpenRouter API [48]. Code for reproducing all experiments is available at http://github.com/Mystic-Slice/LLM-Planning-CodeGen.

## 5. Results

Table 1 shows the average energy collected in the GRASP dataset, averaged over the five energy distributions and both obstacle settings, comparing DG with IR. Across most models (except for DeepSeek-R1), IR outperforms DG. Overall, GPT-o1 and Claude-Sonnet-3.7 show substantial gains, with IR achieving 200% and 114% improvement. Gemini-2.5-Pro exhibits the largest percentage improvement overall, turning negative DG scores (e.g. −0.52) into high positive IR scores (e.g. 3.22), with a gain of 719%. GPT-o3-mini, despite strong baseline performance in DG (e.g. 2.82 average energy), benefits only slightly from IR (a 3% gain). By contrast, GPT-4o shows limited effectiveness, with net energy scores remaining negative even after IR despite a 71% relative gain, while DeepSeek-R1 uniquely exhibits consistent performance declines (a 14% drop), suggesting potential incompatibility with the IR strategy.

**Table 1.** Performance of LLMs on the GRASP benchmark and three MiniGrid tasks: **Unlock**, **Door-Key** and **Unlock-Pickup**. For GRASP, the metric is the average net energy collected and for MiniGrid the average reward, each reported as the mean ± 95% confidence interval of the mean. Each model is tested using two strategies: **direct generation (DG)** and **iterative refinement (IR)**. Blue/red text indicates the percentage improvement or deterioration of IR over DG.

	GRASP		Unlock		Door-Key		Unlock-Pickup	
model	DG	IR	DG	IR	DG	IR	DG	IR
GPT-4o	−1.53 ± 0.06	−0.45 ± 0.05 +71%	0.00 ± 0.00	0.00 ± 0.00 +0%	0.00 ± 0.00	0.00 ± 0.00 +0%	0.00 ± 0.00	0.00 ± 0.00 +0%
GPT-o1	0.94 ± 0.04	2.82 ± 0.05 +200%	0.97 ± 0.00	0.97 ± 0.00 +0%	0.97 ± 0.00	0.97 ± 0.00 +0%	0.95 ± 0.00	0.95 ± 0.00 +0%
GPT-o3-mini	2.82 ± 0.05	2.90 ± 0.04 +3%	0.90 ± 0.02	0.97 ± 0.00 +8%	0.78 ± 0.02	0.98 ± 0.00 +26%	0.00 ± 0.00	0.78 ± 0.02 +∞%
Claude-Sonnet-3.7	1.30 ± 0.05	2.78 ± 0.04 +114%	0.71 ± 0.03	0.90 ± 0.02 +27%	0.75 ± 0.03	0.88 ± 0.02 +17%	0.55 ± 0.03	0.94 ± 0.00 +70%
Gemini-2.5-Pro	−0.52 ± 0.03	3.22 ± 0.05 +719%	0.97 ± 0.00	0.97 ± 0.00 +0%	0.71 ± 0.03	0.90 ± 0.02 +27%	0.89 ± 0.01	0.89 ± 0.01 +0%
DeepSeek-R1	1.85 ± 0.04	1.59 ± 0.03 -14%	0.00 ± 0.00	0.97 ± 0.00 +∞%	0.92 ± 0.01	0.83 ± 0.02 -10%	0.86 ± 0.02	0.86 ± 0.02 +0%

Table 1 also reports the performance of the LLMs on the three MiniGrid tasks (Unlock, Door-Key and Unlock-Pickup), comparing the DG and IR strategies by average reward. Among all models, GPT-o1 consistently shows perfect or near perfect performance under DG, leaving no room for improvement for IR. By contrast, GPT-o3-mini and Claude-Sonnet-3.7 benefit substantially from IR, especially in more complex tasks like Unlock-Pickup. GPT-o3-mini improves from total failure (0.00 reward) to strong performance (0.78 reward), while Claude-Sonnet-3.7 achieves similar gains, with a 70% increase in reward.

Other models show more varied results. Gemini-2.5-Pro displays meaningful improvement only in the Door-Key task, with static performance elsewhere. DeepSeek-R1 shows inconsistent behaviour: IR dramatically increases performance in the Unlock task from 0.00 to 0.97 in reward, yet decreases performance in Door-Key and has no gain on Unlock-Pickup. On the other hand, GPT-4o fails to improve using IR across all tasks, maintaining 0 reward, indicating fundamental issues with task comprehension or execution.

Table 2 provides a comparative analysis of IR against several other prompting and programmatic baselines: CoT, two-step CoT, random and greedy, on the GRASP and MiniGrid tasks using five models.^2^ Importantly, these approaches do not fall under the IPP framework; instead, they operate through natural language prompting and direct action generation without synthesizing executable code. For GRASP, in order to evaluate the different methods on the hardest of tasks, the instances were configured to allow eight movement directions with a carry limit of 2 and a cost per step of 0.3, with the agent starting inside the grid. On the GRASP benchmark, IR is the only one that results in a positive average energy score (0.16 ± 0.04), substantially outperforming CoT (−1.02) and two-step CoT (−1.91). Programmatic baselines such as greedy (−3.61) and random (−4.55) perform even worse. Notably, IR accomplishes this with the lowest API cost among LLM-based methods ($8.0\times 10^{-4}$). While all reported costs are averaged over 100 instances, it is important to note that IR generates a single final Python program that generalizes to all future instances. As a result, its cost does not grow with the number of instances evaluated. Once the program is synthesized through IR, it can be reused without invoking the model again. By contrast, CoT and two-step CoT need to generate new action plans for each individual instance, causing their costs to scale linearly with the number of evaluations.

**Table 2.** Comparison of prompting strategies and baseline methods across five LLMs on the GRASP and MiniGrid benchmarks. Within each model, we report naive prompting, CoT, two-step CoT and IR; random and greedy are model-independent baselines shown once. For GRASP, the metric is net energy collected; for MiniGrid, it is reward (maximum 1.0). Each cell reports the mean and its 95% confidence interval, with the per-instance API cost in USD in parentheses. Results are averaged over 100 instances per task. For GRASP, instances use the hardest configuration: eight movement directions, a carry limit of 2, a cost per step of 0.3 and the agent starting inside the grid.

		GRASP			
model	method	mean ± 95% CI (cost)	Unlock	Door-Key	Unlock-Pickup
baselines	random	−4.55 ± 0.18 (0)	0.00 ± 0.00 (0)	0.00 ± 0.00 (0)	0.00 ± 0.00 (0)
	greedy	−3.61 ± 0.05 (0)	0.97 ± 0.00 (0)	0.98 ± 0.00 (0)	0.75 ± 0.07 (0)
GPT-4o	naive prompting	−1.66 ± 0.29 ($0.0066)	0.09 ± 0.05 ($0.0060)	0.00 ± 0.00 ($0.0070)	0.00 ± 0.00 ($0.0070)
	CoT	−2.12 ± 0.28 ($0.0080)	0.11 ± 0.06 ($0.0080)	0.00 ± 0.00 ($0.0070)	0.00 ± 0.00 ($0.0110)
	two-step CoT	−3.10 ± 0.30 ($0.0146)	0.09 ± 0.05 ($0.0090)	0.02 ± 0.03 ($0.0180)	0.00 ± 0.00 ($0.0160)
	IR	−0.84 ± 0.16 ($0.0018)	0.00 ± 0.00 ($0.0005)	0.00 ± 0.00 ($0.0004)	0.00 ± 0.00 ($0.0005)
GPT-o3-mini	naive prompting	−0.97 ± 0.13 ($0.0018)	0.97 ± 0.00 ($0.0010)	0.96 ± 0.03 ($0.0020)	0.53 ± 0.09 ($0.0020)
	CoT	−1.02 ± 0.15 ($0.0020)	0.97 ± 0.00 ($0.0020)	0.95 ± 0.03 ($0.0030)	0.48 ± 0.09 ($0.0030)
	two-step CoT	−1.91 ± 0.20 ($0.0039)	0.97 ± 0.00 ($0.0020)	0.97 ± 0.02 ($0.0020)	0.84 ± 0.06 ($0.0030)
	IR	0.16 ± 0.04 ($0.0008)	0.97 ± 0.00 ($0.0011)	0.98 ± 0.00 ($0.0008)	0.76 ± 0.07 ($0.0008)
Claude-Sonnet-3.7	naive prompting	−3.72 ± 0.30 ($0.0116)	0.04 ± 0.04 ($0.0050)	0.00 ± 0.00 ($0.0050)	0.00 ± 0.00 ($0.0060)
	CoT	−4.00 ± 0.30 ($0.0115)	0.04 ± 0.04 ($0.0050)	0.00 ± 0.00 ($0.0060)	0.00 ± 0.00 ($0.0060)
	two-step CoT	−3.70 ± 0.25 ($0.0206)	0.05 ± 0.04 ($0.0110)	0.02 ± 0.03 ($0.0130)	0.00 ± 0.00 ($0.0140)
	IR	−0.04 ± 0.06 ($0.0038)	0.91 ± 0.05 ($0.0039)	0.91 ± 0.05 ($0.0016)	0.94 ± 0.00 ($0.0053)
Gemini-2.5-Pro	naive prompting	−0.85 ± 0.17 ($0.0103)	0.93 ± 0.04 ($0.0020)	0.89 ± 0.06 ($0.0020)	0.52 ± 0.09 ($0.0040)
	CoT	−0.95 ± 0.21 ($0.0115)	0.88 ± 0.05 ($0.0090)	0.87 ± 0.06 ($0.0090)	0.55 ± 0.09 ($0.0080)
	two-step CoT	−1.11 ± 0.15 ($0.0136)	0.90 ± 0.05 ($0.0030)	0.76 ± 0.08 ($0.0030)	0.54 ± 0.09 ($0.0050)
	IR	−0.12 ± 0.07 ($0.0019)	0.97 ± 0.00 ($0.0017)	0.91 ± 0.05 ($0.0045)	0.89 ± 0.04 ($0.0026)
DeepSeek-R1	naive prompting	−1.39 ± 0.39 ($0.0018)	0.87 ± 0.06 ($0.0010)	0.85 ± 0.07 ($0.0010)	0.71 ± 0.09 ($0.0010)
	CoT	−1.40 ± 0.37 ($0.0020)	0.76 ± 0.08 ($0.0010)	0.84 ± 0.07 ($0.0010)	0.84 ± 0.06 ($0.0010)
	two-step CoT	−2.70 ± 0.35 ($0.0039)	0.84 ± 0.07 ($0.0020)	0.82 ± 0.07 ($0.0020)	0.77 ± 0.08 ($0.0020)
	IR	−0.65 ± 0.06 ($0.0009)	0.97 ± 0.00 ($0.0008)	0.87 ± 0.06 ($0.0009)	0.87 ± 0.05 ($0.0006)

For the three MiniGrid tasks, IR matches or slightly exceeds the other best performing method. For instance, in the Unlock and Door-Key tasks, IR achieves a perfect or near perfect reward (0.97 and 0.98, respectively), comparable to or better than CoT and two-step CoT, but at a substantially lower API cost (as low as $8.1\times 10^{-4}$ per instance). In the Unlock-Pickup task, IR (0.76±0.07) performs better than CoT (0.48) and nearly matches two-step CoT (0.84), again at a reduced cost. These results suggest that IR offers a better balance of performance and cost than other LLM-based methods, making it practical for complex planning tasks that demand precise execution.

Figure 5 shows the performance of all six LLMs using IR across four tasks: GRASP, Unlock, Door-Key and Unlock-Pickup, with the numerical results presented in table 3. The x-axis shows the number of refinement iterations, with 0 representing DG and higher values indicating additional refinement steps. In the GRASP benchmark, GPT-o1 and GPT-o3-mini reach a peak energy score of 2.82 and 2.90 at iteration 1, while Claude-Sonnet-3.7 peaks at 2.78 at iteration 2 before slightly declining. Among the added models, Gemini-2.5-Pro shows the largest benefit from refinement, climbing from −0.52 at iteration 0 to a peak of 3.22 at iteration 3, whereas GPT-4o remains negative throughout (improving only from −1.53 to −0.45), and DeepSeek-R1 starts relatively strong at 1.85 but declines slightly to 1.59.

**Figure 5.**
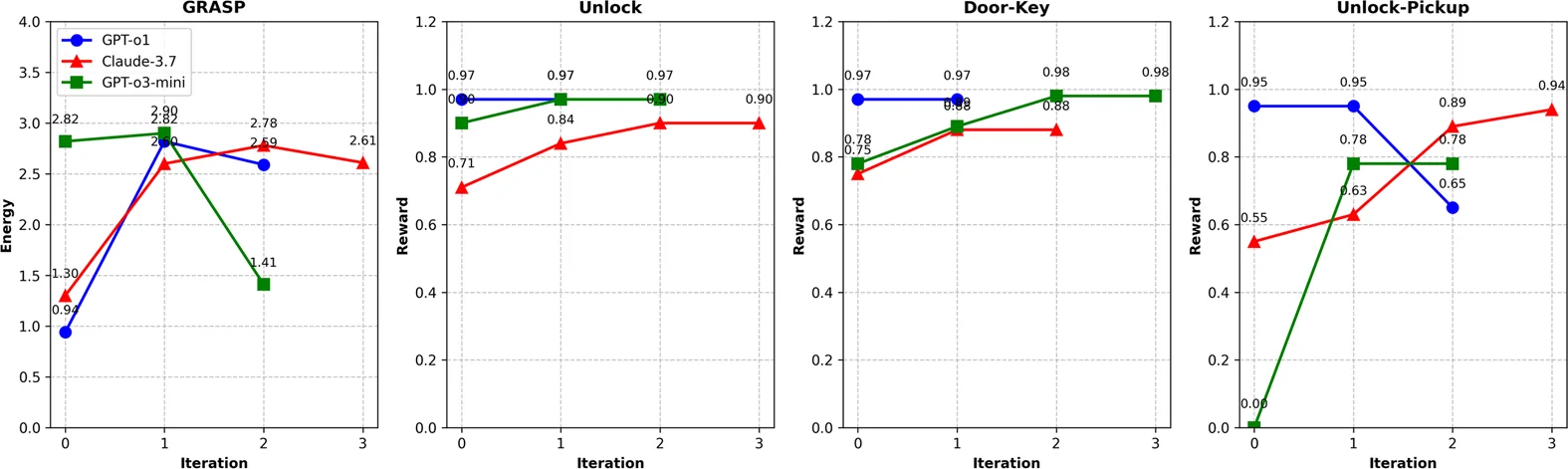
Performance of all six LLMs (GPT-4o, GPT-o1, GPT-o3-mini, Claude-Sonnet-3.7, Gemini-2.5-Pro and DeepSeek-R1) across four tasks—*GRASP*, *Unlock*, *Door-Key* and *Unlock-Pickup*—measured over successive refinement iterations. For GRASP, the metric is energy collected; for MiniGrid tasks, the metric is reward (maximum = 1.0). Iteration 0 corresponds to the initial output produced by the DG method.

**Table 3.** Performance of all six LLMs (GPT-4o, GPT-o1, GPT-o3-mini, Claude-Sonnet-3.7, Gemini-2.5-Pro and DeepSeek-R1) across the four tasks, namely, *GRASP*, *Unlock*, *Door-Key* and *Unlock-Pickup* measured over successive refinement iterations. For GRASP, the metric is energy collected; for MiniGrid tasks, the metric is reward (maximum = 1.0). Iteration 0 corresponds to the initial output produced by the DG method. Each cell is mean ± 95% confidence interval of the mean.

dataset	model	iter 0	iter 1	iter 2	iter 3	iter 4	iter 5
**GRASP (energy)**	GPT-o1	0.94 ± 0.04	2.82 ± 0.05	2.59 ± 0.05	—	—	—
	Claude-Sonnet-3.7	1.30 ± 0.05	2.60 ± 0.04	2.78 ± 0.04	2.61 ± 0.05	—	—
	GPT-o3-mini	2.82 ± 0.05	2.90 ± 0.04	1.41 ± 0.06	—	—	—
	GPT-4o	−1.53 ± 0.06	−1.45 ± 0.06	−1.01 ± 0.05	−0.79 ± 0.06	−0.45 ± 0.05	−0.45 ± 0.05
	Gemini-2.5-Pro	−0.52 ± 0.03	3.10 ± 0.05	3.21 ± 0.05	3.22 ± 0.05	3.01 ± 0.05	—
	DeepSeek-R1	1.85 ± 0.04	1.59 ± 0.03	—	—	—	—
**Unlock (reward)**	GPT-o1	0.97 ± 0.00	0.97 ± 0.00	0.97 ± 0.00	—	—	—
	Claude-Sonnet-3.7	0.71 ± 0.03	0.84 ± 0.02	0.90 ± 0.02	0.90 ± 0.02	—	—
	GPT-o3-mini	0.90 ± 0.02	0.90 ± 0.02	0.97 ± 0.00	0.97 ± 0.00	—	—
	GPT-4o	0.00 ± 0.00	0.00 ± 0.00	—	—	—	—
	Gemini-2.5-Pro	0.97 ± 0.00	0.97 ± 0.00	—	—	—	—
	DeepSeek-R1	0.00 ± 0.00	0.93 ± 0.01	0.97 ± 0.00	0.97 ± 0.00	—	—
**Door-Key (reward)**	GPT-o1	0.97 ± 0.00	0.97 ± 0.00	0.00 ± 0.00	—	—	—
	Claude-Sonnet-3.7	0.75 ± 0.03	0.88 ± 0.02	0.88 ± 0.02	—	—	—
	GPT-o3-mini	0.78 ± 0.02	0.89 ± 0.02	0.98 ± 0.00	0.98 ± 0.00	—	—
	GPT-4o	0.00 ± 0.00	0.00 ± 0.00	—	—	—	—
	Gemini-2.5-Pro	0.71 ± 0.03	0.81 ± 0.02	0.90 ± 0.02	0.00 ± 0.00	—	—
	DeepSeek-R1	0.92 ± 0.01	0.83 ± 0.02	—	—	—	—
**Unlock-Pickup (reward)**	GPT-o1	0.95 ± 0.00	0.95 ± 0.00	0.65 ± 0.01	—	—	—
	Claude-Sonnet-3.7	0.55 ± 0.03	0.63 ± 0.03	0.89 ± 0.01	0.94 ± 0.00	0.94 ± 0.00	—
	GPT-o3-mini	0.00 ± 0.00	0.78 ± 0.02	0.78 ± 0.02	—	—	—
	GPT-4o	0.00 ± 0.00	0.00 ± 0.00	—	—	—	—
	Gemini-2.5-Pro	0.89 ± 0.01	0.89 ± 0.01	—	—	—	—
	DeepSeek-R1	0.86 ± 0.02	0.86 ± 0.02	—	—	—	—

In the three MiniGrid tasks, GPT-o1 and GPT-o3-mini perform well from the beginning, often achieving near perfect scores by iteration 1. Claude-Sonnet-3.7 shows a meaningful improvement, particularly in the Unlock and Unlock-Pickup tasks, where rewards increase from 0.71 to 0.90 and from 0.55 to 0.94, respectively. Compared to GRASP, performance in the MiniGrid tasks declines less often with additional refinement. The added models reinforce this picture but also expose its limits. DeepSeek-R1 improves sharply on Unlock, rising from 0.00 to 0.97, whereas GPT-4o fails to make any progress on the MiniGrid tasks, remaining at zero throughout. Refinement is not always monotonic, however: Gemini-2.5-Pro reaches a reward of 0.90 on Door-Key by iteration 2 but then regresses to 0.00 at iteration 3, showing that an additional refinement step can occasionally overwrite a previously correct program. Overall, the results show that most performance gains occur within the first one or two iterations of IR. Beyond that point, improvements tend to level off or slightly decrease.

As noted in §4.2, the pseudocode extension and step-by-step strategies are evaluated only on the GRASP benchmark. Figure 6 presents two analyses of performance on the GRASP task using all six LLMs, with corresponding numerical results provided in table 4. On the left, it shows the comparison between the greedy programmatic baseline and the pseudocode extension. GPT-o1, which performs reasonably well under the greedy baseline, fails under pseudocode extension with a negative energy score. On the other hand, Claude-Sonnet-3.7 and GPT-o3-mini both benefit from this structured guidance, achieving higher energy scores with pseudocode extension, indicating that models with stronger capabilities are better at exploiting high-level pseudocode instructions. The added models sharpen this trend: DeepSeek-R1 and Gemini-2.5-Pro obtain the largest gains from pseudocode extension (3.28 and 2.87, up from the greedy baseline of 0.89), whereas the weaker GPT-4o, like GPT-o1, is hurt by it and drops to −1.69. On the right, step-by-step performance is shown from step 0 to step 4 when the complexity of the GRASP environment is gradually added. GPT-o1 shows rapid improvement, peaking at step 2 (2.13) before dropping at step 3, then recovering at step 4. Claude-Sonnet-3.7 and GPT-o3-mini show steadier gains, with Claude-Sonnet-3.7 peaking at step 2 and declining at step 3, while GPT-o3-mini shows consistent upward trends. This step-2 local peak is shared by several models: GPT-o1, Claude-Sonnet-3.7 and DeepSeek-R1 all reach a local maximum at step 2 and then dip at step 3 before partially recovering at step 4, suggesting that each newly introduced constraint can temporarily make the problem harder and push the models towards suboptimal solutions until they adapt. GPT-4o, by contrast, never adapts and stays near −2 across all steps, while Gemini-2.5-Pro improves almost monotonically once it clears its initially negative scores. These results suggest that incremental exposure to complexity helps models adapt, though performance can fluctuate depending on how well a model generalizes under new constraints.

**Figure 6.**
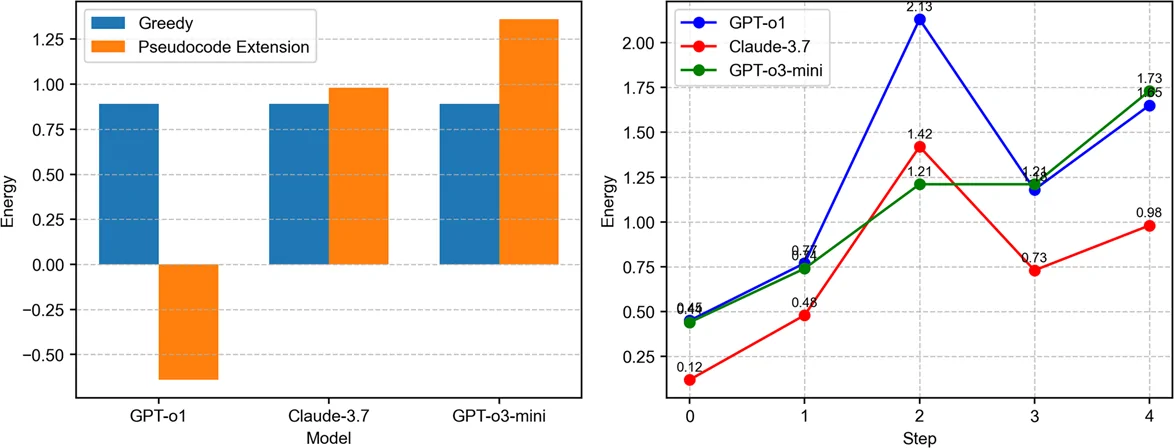
Left: performance comparison of *greedy* and *pseudocode extension* strategies on the GRASP task, evaluated across all six LLMs (GPT-4o, GPT-o1, GPT-o3-mini, Claude-Sonnet-3.7, Gemini-2.5-Pro and DeepSeek-R1). The results highlight the performance gains enabled by pseudocode-guided reasoning. Right: step-by-step performance on GRASP, where environment complexity is introduced incrementally over four steps.

**Table 4.** Energy comparison by model, approach and step. The table compares greedy, pseudocode extension (PE), iterative refinement (IR) and the intermediate codes from the different steps produced by step-by-step methodology. Only for GRASP. Each cell is mean ± 95% confidence interval of the mean.

model	greedy	PE	step 0	step 1	step 2	step 3	step 4	IR
GPT-4o	0.89 ± 0.05	−1.69 ± 0.04	−2.04 ± 0.05	−2.03 ± 0.05	−2.03 ± 0.05	−2.03 ± 0.05	−2.00 ± 0.05	−0.45 ± 0.05
GPT-o1	0.89 ± 0.05	−0.64 ± 0.03	0.45 ± 0.06	0.77 ± 0.06	2.13 ± 0.06	1.18 ± 0.03	1.65 ± 0.03	2.82 ± 0.05
GPT-o3-mini	0.89 ± 0.05	1.36 ± 0.06	0.44 ± 0.04	0.74 ± 0.04	1.21 ± 0.05	1.21 ± 0.05	1.73 ± 0.05	2.90 ± 0.04
Claude-Sonnet-3.7	0.89 ± 0.05	0.98 ± 0.05	0.12 ± 0.06	0.48 ± 0.06	1.42 ± 0.05	0.73 ± 0.02	0.98 ± 0.02	2.78 ± 0.04
Gemini-2.5-Pro	0.89 ± 0.05	2.87 ± 0.06	−0.51 ± 0.05	−0.46 ± 0.05	0.63 ± 0.05	1.62 ± 0.03	1.79 ± 0.03	3.22 ± 0.05
DeepSeek-R1	0.89 ± 0.05	3.28 ± 0.05	−1.23 ± 0.06	−1.23 ± 0.06	−0.98 ± 0.06	−1.50 ± 0.04	0.00 ± 0.00	1.85 ± 0.04

Taken together, these results offer practical guidance on which strategy to use and when. Across both benchmarks, IR is the most reliable default: it is the only method that yields a positive average energy on the hardest GRASP configuration, and it matches or exceeds every non-code baseline on MiniGrid, while its cost is effectively amortized to zero because it produces a single reusable program rather than replanning for each instance. Since almost all of its benefit arrives within the first one or two iterations, we recommend capping refinement at a small budget; additional iterations rarely help and can occasionally overwrite an already-working program. Method choice, however, interacts strongly with model capability. For strong reasoning models such as GPT-o1 and GPT-o3-mini, DG already solves the simpler tasks, so refinement mainly matters on the harder ones such as Unlock-Pickup, whereas the largest IR gains instead accrue to mid-capability models such as Claude-Sonnet-3.7. Pseudocode extension is worth using when a reasonable greedy skeleton is available and the underlying model is capable enough to exploit high-level structure, as with Claude-Sonnet-3.7, GPT-o3-mini, Gemini-2.5-Pro and DeepSeek-R1, but it can hurt models that follow the scaffold too literally, such as GPT-o1 and GPT-4o. The step-by-step curriculum is most useful when the target environment is complex, since incremental exposure to constraints helps models adapt, though performance can fluctuate as each new constraint is introduced. Finally, the non-code baselines, CoT and two-step CoT, are the least attractive option whenever a code policy can be synthesized: they are weaker on the harder tasks and, unlike IR, their cost grows linearly with the number of instances evaluated. One caveat cuts across all of these choices: no strategy rescued GPT-4o, which failed to comprehend the tasks well enough for any prompting method to help, underscoring that a minimum level of model capability is a prerequisite rather than something the framework can supply.

## 6. Case study

To further understand the qualitative difference between the different methods, we examine representative instances where the proposed IPP framework succeeds, while the other baselines from table 2 underperform or fail.

### 6.1. GRASP

In this section, we conduct a detailed case study of a representative sample instance (grid index 9 from the inner spiral block configuration) GRASP task, shown in figure 7. This instance is particularly informative because it requires the agent to carefully balance exploration, energy collection and movement efficiency under explicit cost and capacity constraints. Table 5 summarizes the action sequences produced by the *GPT-o3-mini* model when solving this instance using a variety of prompting-based baselines and programmatic planning strategies.

**Figure 7.**
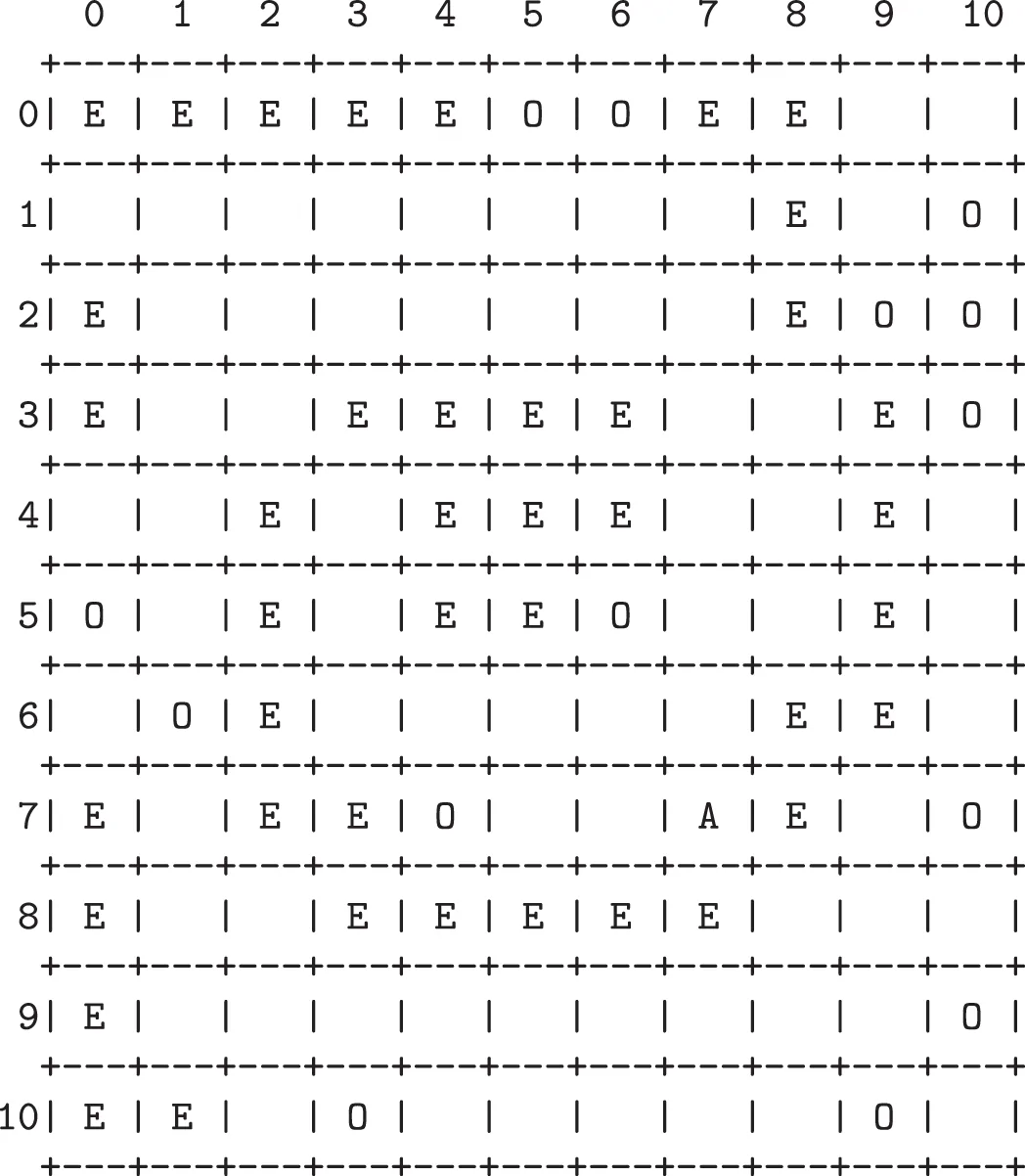
GRASP grid instance (grid index 9 from the inner spiral block configuration) with best solution by the IPP framework while other baselines from table 2 underperform.

**Table 5.** Comparison of prompting strategies, baseline and programmatic methods on the sample instance of the GRASP task using *GPT-o3-mini*. This instance was configured to allow all eight directions of movement for the agent with a cost per step of 0.3 and a carry limit of two energy tokens.

method	actions	final energy	reason for performance gap
naive prompting	‘RIGHT’, ‘TAKE’, ‘UP’, ‘TAKE’, ‘DOWNLEFT’, ‘DROP’, ‘DROP’, ‘DOWN’, ‘TAKE’, ‘LEFT’, ‘TAKE’, ‘UPRIGHT’, ‘DROP’, ‘DROP’, ‘UPRIGHT’, ‘RIGHT’, ‘TAKE’, ‘DOWNLEFT’, ‘LEFT’, ‘DROP’	−1	performed a ’DROP’ action for each energy token carried although only one ’DROP’ is necessary to drop all the energy tokens. Failed to account for cost per step and therefore wasted energy in movement
CoT	’RIGHT’, ‘TAKE’, ‘UP’, ‘TAKE’, ‘DOWNLEFT’, ‘DROP’, ‘DROP’, ‘DOWN’, ‘TAKE’, ‘LEFT’, ‘TAKE’, ‘UPRIGHT’, ‘DROP’, ‘DROP’, ‘UPRIGHT’, ‘RIGHT’, ‘TAKE’, ‘LEFT’, ‘DOWNLEFT’, ‘DROP’	−1	performed a ’DROP’ action for each energy token carried although only one ’DROP’ is necessary to drop all the energy tokens. Failed to account for cost per step and therefore wasted energy in movement
two-step CoT	’RIGHT’, ‘TAKE’, ‘LEFT’, ‘DROP’, ‘UPRIGHT’, ‘TAKE’, ‘DOWNLEFT’, ‘DROP’, ‘LEFT’, ‘LEFT’, ‘DOWN’, ‘TAKE’, ‘RIGHT’, ‘TAKE’, ‘UPLEFT’, ‘DROP’, ‘DROP’	−3.1	lost track of the starting position where the energy tokens had to be dropped
DG	’UPRIGHT’, ‘TAKE’, ‘DOWN’, ‘TAKE’, ‘LEFT’, ‘DROP’, ‘DOWNLEFT’, ‘TAKE’, ‘RIGHT’, ‘TAKE’, ‘UP’, ‘DROP’, ‘UPLEFT’, ‘UPLEFT’, ‘TAKE’, ‘DOWNRIGHT’, ‘DOWNRIGHT’, ‘DROP’	−0.4	failed to account for cost per step and therefore wasted energy in movement
IR	’DOWNLEFT’, ‘TAKE’, ‘RIGHT’, ‘TAKE’, ‘UP’, ‘DROP’, ‘UPRIGHT’, ‘TAKE’, ‘DOWN’, ‘TAKE’, ‘LEFT’, ‘DROP’	0.4	N/A

While the baseline methods are often able to identify valid local actions such as collecting nearby energy tokens, they fail to optimize for the cost-per-step constraint. As a result, their generated solutions incur excessive movement and redundant actions, ultimately leading to negative net energy at the end of the episode. In particular, both the naive and CoT prompting strategies repeatedly perform unnecessary DROP actions—one per carried energy token—despite the environment requiring only a single DROP to unload all carried items. This inefficiency, combined with unplanned navigation, significantly degrades overall performance.

DG exhibits a modest improvement over pure prompting-based approaches by producing somewhat shorter and more coherent trajectories. Nevertheless, it still fails to explicitly reason about the cumulative movement cost, resulting in avoidable energy loss that prevents it from achieving a positive final energy balance.

The limitations of LLMs in maintaining consistent state representations are most evident in the two-step CoT strategy. The model loses track of the agent's original drop-off location after several steps. This breakdown in state management causes the plan to derail midway through execution, leading an incorrect termination state with substantial energy loss.

By contrast, the IR strategy successfully adheres to the task constraints throughout the episode. By explicitly refining its plan and accounting for both movement costs and carry limits, IR produces a concise and goal-directed sequence of actions that avoids redundant operations and limits unnecessary exploration. As a result, it is the only method in this comparison that consistently terminates with a positive net energy collected.

To make the utility of the refinement mechanism concrete, we trace how the GPT-o3-mini program evolves across successive IR iterations on this instance; figure 8 shows the token-selection core of the synthesized program at each step. The initial program (equivalent to DG, the DG row of table 5) follows a short-sighted collection strategy (figure 8a) and, crucially, never weighs the round-trip movement cost against the value of a token, so it terminates with negative net energy.

**Figure 8.**
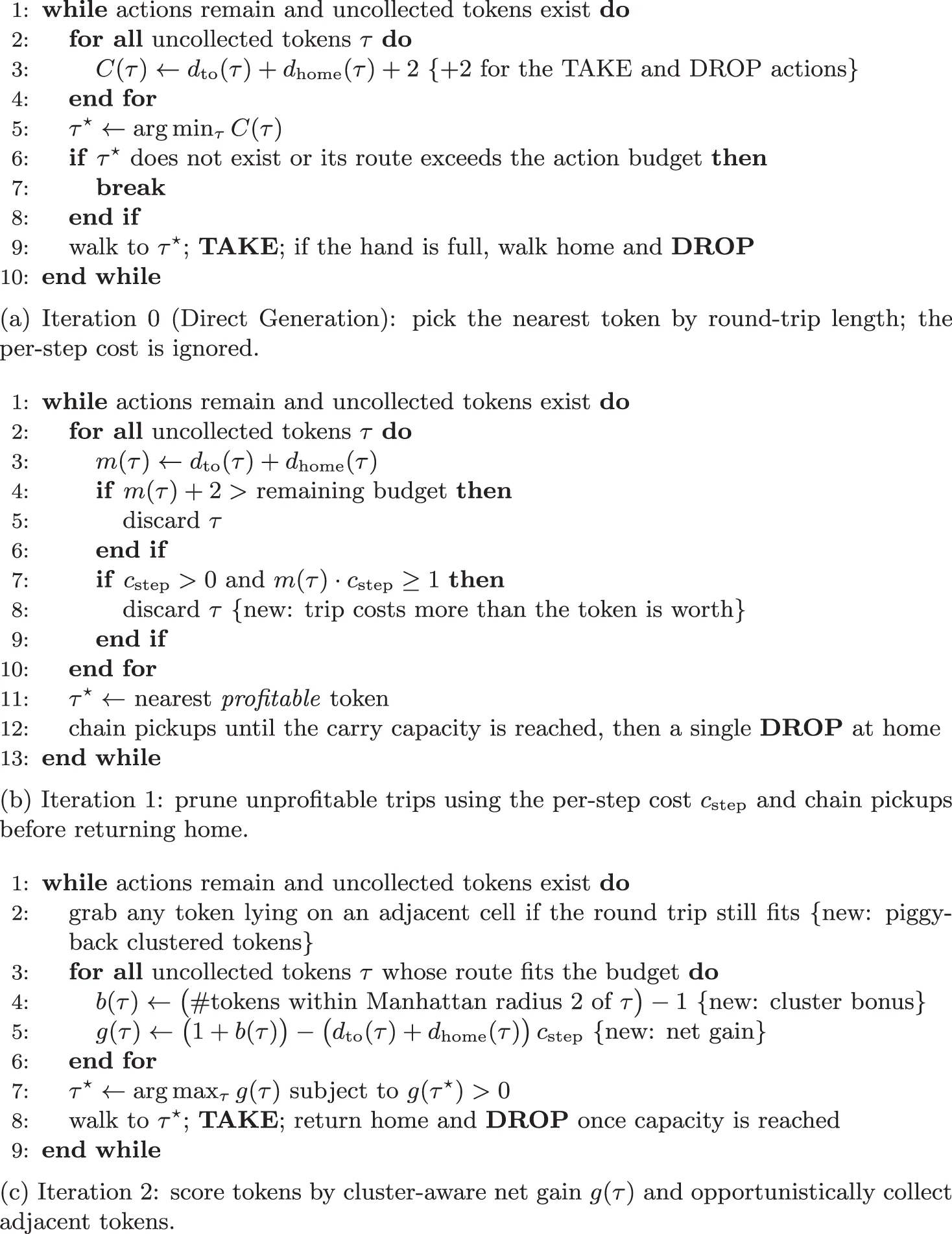
Evolution of the GRASP token-selection heuristic in the GPT-o3-mini program across IR steps, on the instance of figure 7. Only the token-selection core is shown; the breadth first search shortest-path routine, grid parsing and action-budget bookkeeping are identical across iterations. Here, $d_{\mathrm{to}}(\tau )$ and $d_{\mathrm{home}}(\tau )$ are the number of steps from the agent's current cell to token τ and from τ back to the start cell, and $c_{\mathrm{step}}$ is the per-step energy cost. Lines marked ‘new’ are those introduced at that iteration.

At the second iteration, after being shown its worst-performing grids as feedback, the model explicitly decides to ‘think ahead’ rather than collect one token at a time. It revises the code so that the agent strings together several profitable pickups in a single excursion—accounting for its carry capacity, the number of moves needed to reach a token and return home, and the per-step cost—before making a single DROP back at the start cell. In the same step, it adds a pruning rule that discards any move whose travel cost would exceed the single energy unit a token is worth (figure 8b). This directly targets the failure mode responsible for the negative initial score, namely, movement that is not justified by the energy it collects.

At the third iteration, the model refines this ‘greedy-chain’ strategy further with two changes (figure 8c). First, it opportunistically ‘piggy-backs’ tokens that are immediately adjacent to the current cell whenever picking them up and still returning home remains feasible, so that the cost of extra moves is amortized across clustered tokens. Second, it replaces its next-token heuristic with an explicit scoring function that favours candidates lying in dense regions. Each candidate token τ is scored by a net gain

(6.1) $$g(\tau )=(1+b(\tau ))-(d_{\mathrm{to}}(\tau )+d_{\mathrm{home}}(\tau ))\;c_{\mathrm{step}},$$

where b(τ) is a cluster bonus equal to the number of other tokens lying within a Manhattan radius of 2 of τ, $d_{\mathrm{to}}(\tau )$ and $d_{\mathrm{home}}(\tau )$ are the number of steps from the agent's current cell to τ and from τ back to the start cell respectively, $c_{\mathrm{step}}$ is the per-step energy cost and the leading constant is the single unit of energy that the token itself is worth. A token is considered only if its net gain is positive, g(τ)>0, and among candidates with positive gain the one requiring the fewest total steps is chosen; the model additionally verifies that every planned route, including its TAKE and DROP actions, fits within the remaining action budget. The resulting program produces the concise, 12-action trajectory shown in the IR row of table 5, the only solution in this comparison to finish with positive net energy. This progression illustrates the core of the IR loop in figure 2: at each step, the model is confronted with concrete failure cases and converts them into a targeted, verifiable change in the synthesized code. This case study highlights the importance of structured, iterative planning mechanisms for enforcing global constraints and maintaining accurate state representations in sequential decision-making tasks.

### 6.2. MiniGrid (Unlock-Pickup)

In this section, we analyse a representative sample instance of the MiniGrid Unlock-Pickup task (grid seed 28), illustrated in figure 9. The action sequences generated by the *GPT-o3-mini* model under different methods are summarized in table 6.

**Figure 9.**
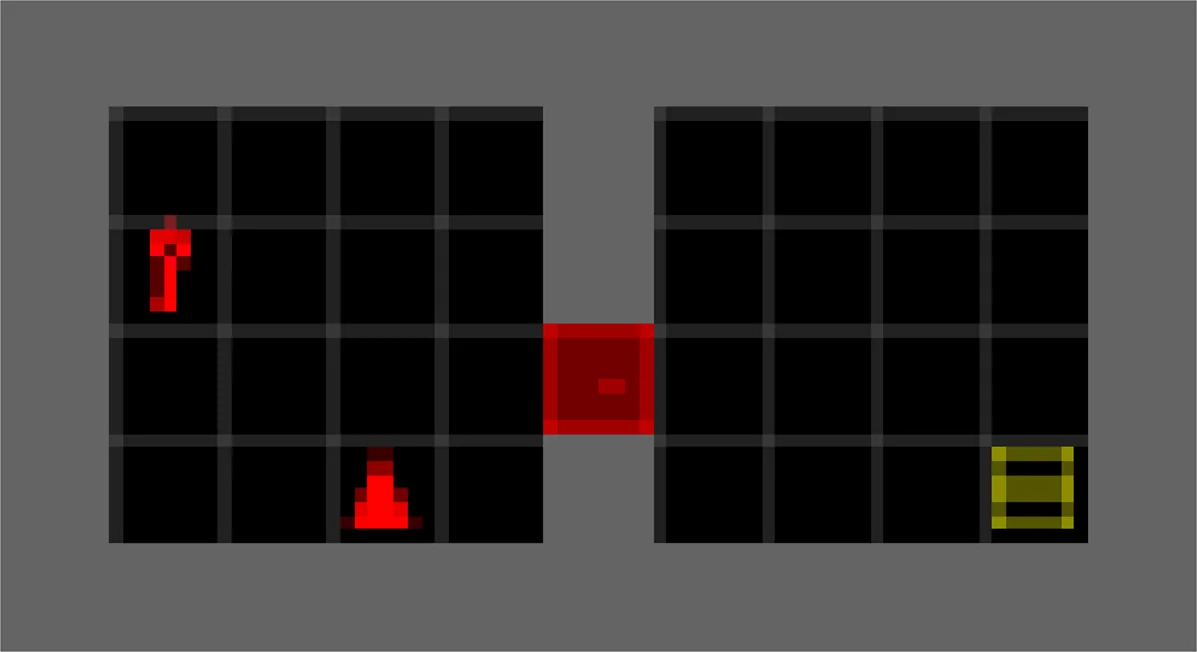
Sample instance (grid seed 28) of the MiniGrid Unlock-Pickup task solved by the IPP framework while unsolved by other baselines from table 2.

**Table 6.** Comparison of prompting strategies, baseline and programmatic methods on the sample instance (grid seed 28) of the MiniGrid Unlock-Pickup task using *GPT-o3-mini*.

method	actions	success	reason for failure
naive prompting	’MOVE’, ‘MOVE’, ‘LEFT’, ‘MOVE’, ‘PICKUP’, ‘LEFT’, ‘MOVE’, ‘LEFT’, ‘MOVE’, MOVE’, ‘UNLOCK’, ‘MOVE’, ‘MOVE’, ‘MOVE’, ‘MOVE’, ‘MOVE’, ‘RIGHT’, ‘PICKUP’	no	forgot to drop the key before picking up the box
CoT	’MOVE’, ‘MOVE’, ‘LEFT’, ‘MOVE’, ‘PICKUP’, ‘LEFT’, ‘MOVE’, ‘LEFT’, ‘MOVE’, ‘MOVE’, ‘UNLOCK’, ‘MOVE’, ‘MOVE’, ‘MOVE’, ‘MOVE’, ‘RIGHT’, ‘MOVE’, ‘LEFT’, ‘PICKUP’	no	forgot to drop the key before picking up the box
two-step CoT	’MOVE’, ‘MOVE’, ‘LEFT’, ‘MOVE’, ‘PICKUP’, ‘LEFT’, ‘LEFT’, ‘MOVE’, ‘MOVE’, ‘RIGHT’, ‘MOVE’, ‘LEFT’, ‘UNLOCK’, ‘DROP’, ‘MOVE’, ‘MOVE’, ‘MOVE’, ‘MOVE’, ‘MOVE’, ‘RIGHT’, ‘PICKUP’	no	dropped the key on the cell connecting the two rooms and hence unable to move forward
DG	’MOVE’, ‘MOVE’, ‘MOVE’, ‘LEFT’, ‘MOVE’, ‘MOVE’, ‘LEFT’, ‘PICKUP’, ‘LEFT’, ‘MOVE’, ‘RIGHT’, ‘MOVE’, ‘MOVE’, ‘LEFT’, ‘MOVE’, ‘MOVE’, ‘UNLOCK’, ‘MOVE’, ‘MOVE’, ‘MOVE’, ‘MOVE’, ‘MOVE’, ‘RIGHT’, ‘RIGHT’, ‘RIGHT’, ‘DROP’, ‘PICKUP’	no	navigated properly to the box and dropped the key in a nearby empty cell but forgot to turn back to face the box before picking it up
IR	’MOVE’, ‘LEFT’, ‘MOVE’, ‘MOVE’, ‘RIGHT’, ‘PICKUP’, ‘RIGHT’, ‘MOVE’, ‘MOVE’, ‘MOVE’, ‘UNLOCK’, ‘MOVE’, ‘MOVE’, ‘MOVE’, ‘MOVE’, ‘MOVE’, ‘RIGHT’, ‘RIGHT’, ‘RIGHT’, ‘DROP’, ‘RIGHT’, ‘RIGHT’, ‘PICKUP’	yes	N/A

When using naive prompting and standard CoT reasoning, the model is able to navigate through the environment and unlock the door successfully. However, both methods exhibit a fundamental failure in constraint handling: the agent attempts to pick up the box while still carrying the key. Since the environment enforces a single-object carry constraint, this oversight causes the episode to terminate unsuccessfully. These failures indicate that, despite producing seemingly coherent action sequences, the prompting-based methods struggle to consistently enforce object-interaction constraints across longer action horizons.

The two-step CoT strategy demonstrates a partial improvement over the earlier baselines by correctly identifying the need to drop the key before attempting to pick up the box. However, this improvement is undermined by a critical spatial reasoning error. The model drops the key on the narrow cell that connects the two rooms, effectively blocking the only passage required to reach the box. As a result, the agent becomes unable to proceed, illustrating a common failure mode in which local correctness (dropping the key) is achieved at the expense of global feasibility (maintaining navigable paths).

DG further improves upon this behaviour by producing a more globally coherent navigation strategy. In this case, the agent successfully reaches the vicinity of the box and drops the key in a nearby empty cell, avoiding the blockage issue observed in two-step CoT. Despite this progress, DG still fails to complete the task owing to a subtle but crucial execution error where the agent does not reorient itself to face the box before issuing the PICKUP action. This highlights a limitation in handling orientation-dependent actions, even when higher-level planning is largely correct.

Finally, the IR approach resolves all of the above issues and successfully completes the task. By iteratively correcting errors in earlier action sequences, IR ensures that object-handling constraints, spatial layout and agent orientation are all simultaneously satisfied. The resulting plan correctly drops the key in a safe location, navigates back to the box, reorients the agent appropriately and completes the pickup without violating any constraints. This case study underscores the value of iterative, programmatic refinement in addressing compounding errors that arise from long-horizon planning, particularly in environments with strict interaction and orientation constraints.

As with GRASP, we trace how the GPT-o3-mini program changes under refinement on this instance; figure 10 shows the relevant drop-key/pickup-box endgame. The DG program (the DG row of table 6) navigates to the box and drops the key in a nearby empty cell but then issues the final PICKUP without reorienting: because the preceding DROP leaves the agent facing the drop cell rather than the box, the pickup targets the wrong cell and the task fails (figure 10a). After being shown this failure, the refinement makes two targeted changes (figure 10b). First, it reorients the agent to face the box before the final PICKUP, which is the decisive fix for this instance. Second, it selects a drop cell whose direction differs from that of the box, so that the key is never placed on the cell the agent must occupy or pass through—removing the passage-blocking failure exhibited by weaker baselines such as two-step CoT. Together, these two edits convert the DG failure into the successful IR trajectory shown in table 6.

**Figure 10.**
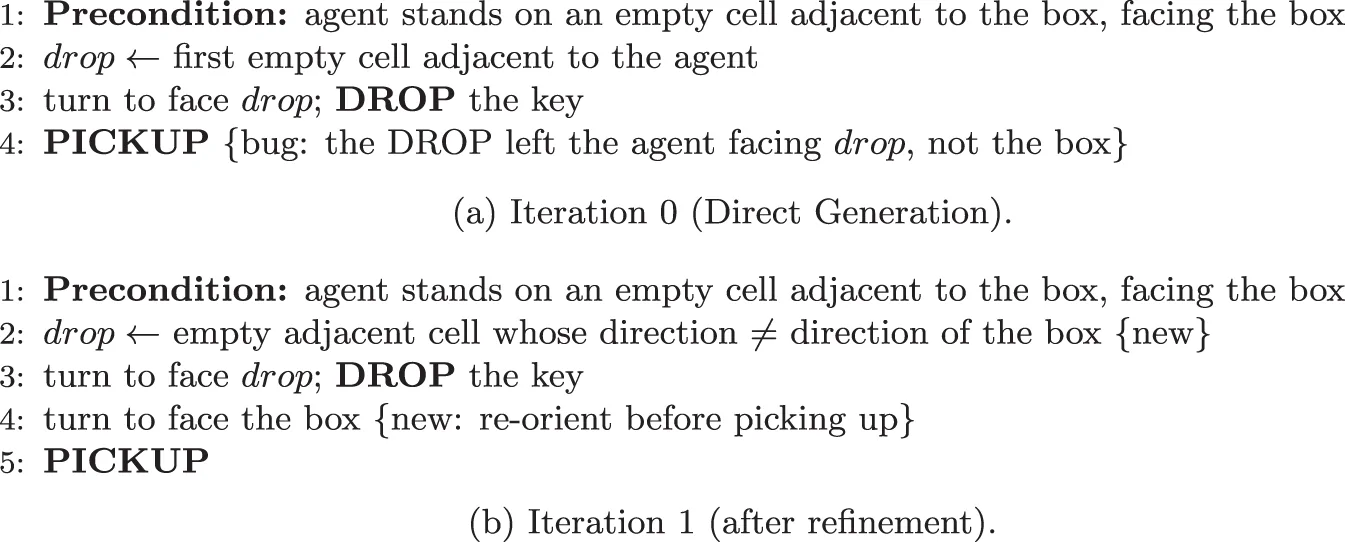
Evolution of the drop-key/pickup-box endgame in the GPT-o3-mini MiniGrid program, on the instance of figure 9. (a) DG drops the key and immediately issues PICKUP, leaving the agent facing the drop cell rather than the box. (b) After refinement, the program chooses a drop cell that is not in the box's direction and reorients to face the box before the pickup. The navigation, breadth first search and rotation routines are identical across iterations and are omitted; lines marked ‘new’ are those introduced by refinement.

## 7. Discussion and limitations

Our experiments show that LLMs, when guided by a structured iterative process, can generate interpretable and reusable code-based planning policies, even on problems they find initially challenging. The proposed IPP framework consistently improves task performance on both GRASP and MiniGrid benchmarks. Even models with a weak initial program benefit significantly from refinement, highlighting the value of execution-based feedback. By treating code as the policy representation, the agent can offload low-level responsibilities such as inventory management, environment state interpretation and management, orientation and path planning onto deterministic Python routines. This separation of concerns allows the LLM to focus its capacity on synthesizing higher level task logic rather than repeatedly rederiving basic operational details. As a result, the model does not need to remember or reason about aspects of the environment that can be precisely encoded and executed by the program. Instead, it can concentrate on designing control flow, conditional structures and decision-making heuristics that constitute the actual strategy. Over successive refinement iterations, this structure provides a stable foundation on which the model can iteratively improve the policy, since deterministic code components ensure consistent behaviour across executions while the LLM adjusts only the parts of the program directly related to strategic reasoning. This leads to more reliable improvements, reduces variance introduced by free form natural language reasoning and supports clearer attribution of errors to either the code's logic or the model's synthesis choices.

Our results demonstrate that both the structure of prompts and the choice of LLMs are critical to planning performance. While direct code generation works in simple scenarios, structured prompting strategies, such as pseudocode extension and curriculum-based step-by-step prompting, significantly improve outcomes on more complex tasks. For IR, most performance gains emerge within the first one or two refinement steps, indicating that the process is effective and relatively computationally efficient. However, this also reveals a limitation of the approach: additional refinement iterations tend to yield diminishing returns. Once the core logic and major failure modes of the program are corrected, further improvements become increasingly incremental. This suggests that IPP is most effective at correcting high-level structural errors and missing heuristics, but may be less suited for fine-grained optimization or subtle edge cases that require highly targeted feedback or domain-specific tuning. In practice, this means that while a small number of refinement steps can substantially improve performance, extended refinement cycles may not be cost-effective and should be applied selectively.

LLMs' response to IR also varies across models. GPT-o1, Claude-Sonnet-3.7 and Gemini-2.5-Pro consistently benefit from the proposed framework, whereas DeepSeek-R1 and GPT-4o sometimes show degraded performance. This variability indicates that IPP inherits model-specific inductive biases and limitations, and that not all LLMs are equally capable of leveraging execution-based feedback to meaningfully revise previously generated code.

To understand why GPT-4o and DeepSeek-R1 do not benefit from IPP, we inspected the programs they produced at each refinement step. The two models fail for very different reasons. GPT-4o's difficulty is conceptual: its initial programs encode an incorrect model of the environment, and refinement never repairs this underlying misconception. On GRASP, it treats energy collection as a purely greedy nearest-first traversal and never weighs the reward of a token against the movement cost of reaching it, so even its converged programs spend more energy moving than they recover and stay net-negative. On the MiniGrid tasks, it repeatedly assumes that the agent can interact with an object while standing on the object's own cell, rather than from an adjacent cell while facing it, so its planned paths step onto keys and doors that are in fact impassable. Successive refinements only adjust surface details such as turn selection or door traversal, and in one case leave the target object unreachable altogether, so GPT-4o never scores above zero on these tasks. IR can amplify competence a model already has, but it cannot supply a correct environment model that the base model lacks.

DeepSeek-R1 fails for a different reason: its programs are largely well structured, but the model does not translate the provided failure instances into productive edits. When asked to refine, it tends to over-constrain an already-working program or add machinery that does not function. On GRASP, its refinement discards a clean collection heuristic in favour of an overly aggressive filter that rejects tokens that are only worth collecting in groups, together with inert scaffolding that is never exercised, and as a result it collects fewer tokens and regresses. On the Door-Key task, its refinement tightens the movement rules to the point of blocking cells the agent legitimately needs, lowering its reward. Rather than diagnosing the specific behaviour responsible for a failure, its revisions restrict or complicate the logic in ways only loosely related to the observed errors. This limited ability to adapt to failure feedback is consistent with the coarse feedback signal discussed above, which does not indicate which part of a program is responsible for a given failure. This behaviour may also stem from the model's training paradigm: there have been claims in the community that reinforcement learning with verifiable rewards (RLVR) does not actually teach the model new knowledge but instead prunes the model's answer space to a narrow subset of correct answers [49–51]. Wu *et al.* [51] note that RLVR involves a trade-off between precision and comprehensive reasoning coverage. These observations could explain why specifically DeepSeek-R1, known to be trained using the RLVR training paradigm, underperforms when used with IPP. While GPT-o1 and GPT-o3-mini are also reasoning models, they are speculated to have been trained using an extended CoT approach with the proximal policy optimization algorithm, which still preserves the model's ability to come up with diverse solutions for a question. Thereby, these models are able to adapt and improve their code upon seeing the failure samples, showing greater flexibility.

A major advantage of IPP is the interpretability of its code-driven policies. Unlike black-box planners or RL agents, the generated programs are human-readable and editable, allowing experts to inspect, debug or adapt plans (see supplementary material, appendix B, for examples). This transparency is particularly valuable in safety-critical domains such as robotics, where understanding and verifying agent behaviour is essential. However, this interpretability benefit is not universal. While code offers deterministic and precise semantics, it may be less accessible to users without programming expertise. For non-technical stakeholders, natural language plans or visual representations may be easier to understand than procedural code. As a result, the interpretability advantages of IPP are currently most pronounced for users with a technical background. Bridging this gap, for example, by automatically generating natural language explanations or higher-level summaries of the synthesized programs, could be a natural direction for future work.

IPP is also more efficient than traditional prompting methods. While CoT prompting requires inference for each instance, IPP produces a single reusable program, reducing API costs and inference latency. Rather than competing directly with RL, our aim is to provide a lower-cost, interpretable alternative that can solve similar planning tasks without intensive environment interaction, making it suitable for real-time or resource-constrained applications.

One other potential limitation of our framework is that it depends heavily on the language model's ability to understand the environment and task rules. While the grid-based set-up is relatively simple, models can still make mistakes in tracking the agent's state, recognizing environment-specific entities or following task constraints. These errors may become more pronounced in more complex environments or when the model encounters novel situations. Although IR can correct some of these issues, there is no guarantee that the final synthesized code will be correct or robust in all cases, a limitation that also applies to RL-based policies.

Another limitation is that the feedback provided to the model during refinement is relatively coarse, typically consisting of aggregate performance metrics or a small set of failure cases. The model is not explicitly informed about which parts of the code are responsible for specific errors, which can make the refinement process less targeted and potentially less efficient when small implementation details have a large impact on performance. We expect this to become a more serious bottleneck as environments grow in scale and horizon. In the small, fully observable grids studied here, a single aggregate score per task carries enough information for the model to infer roughly what went wrong and rewrite the relevant logic. As the state space widens and success depends on longer sequences of interdependent decisions, the same scalar becomes a progressively weaker signal: a lone reward value cannot indicate which of the many intermediate choices caused a failure, so credit assignment becomes harder and refinement is more likely to stall, thrash between competing fixes or overfit to the handful of failure cases it happens to be shown. Settings with sparse or delayed reward, where a policy only pays off after a long correct trajectory, are especially likely to expose this weakness, as are partially observable environments in which the outcome depends on state the program never directly sees. In such regimes, we would expect the coarse signal that suffices in our benchmarks to be insufficient on its own, motivating the richer, more localized feedback signals we discuss as future work.

## 8. Future work

While the proposed IPP framework demonstrates strong empirical performance and favourable amortized cost properties on grid-based benchmarks, several important directions remain open for future investigation.

### 8.1. Partially observable and noisy environments

A primary direction to explore is extending IPP beyond fully observable, static grid worlds to environments with richer dynamics. Although grid-based domains are a foundational abstraction for many planning problems, especially in robotics, real-world settings often involve temporal constraints, stochastic transitions and partial observability. Adapting IPP to partially observable environments would primarily require modifying the feedback provided during IR. The modification could be as simple as the LLM receiving full execution traces describing how the environment evolved in response to actions. This richer feedback could enable the model to revise heuristics and control logic to account for hidden state and observation noise. Evaluating IPP under partial observability, sensor noise and non-deterministic dynamics broadens the applicability and utility of the technique.

### 8.2. More complex planning domains

Another direction to explore is to apply IPP to a broader range of planning problems, including larger grids, more complex constraint structures and domains that combine grid-based planning with temporal or resource constraints. Systematically stress testing IPP in domains of increasing scale and complexity would help clarify its practical limits and its comparative advantages over per instance prompting or RL approaches. Moreover, evaluating the methodology on more complex tasks/games like Minecraft [52] would point out the ceiling of complexity of problems to which IPP is applicable.

### 8.3. Model- and training-aware extensions

Performance differences across LLMs suggest that compatibility with IPP depends on the model used. As can be seen in table 1, reasoning models, namely, GPT-o1, GPT-o3-mini and DeepSeek-R1, tend to improve on their DG code only slightly, if at all, when used with the IPP methodology in comparison to the other models. Future work could systematically study how training paradigms affect iterative code refinement and explore model-aware prompting or refinement strategies that adapt to these differences. More ambitiously, IR itself could be incorporated into post-training or fine-tuning, allowing models to explicitly learn how to interpret failure cases and update code accordingly. Because code outputs are executable and verifiable, they provide a natural substrate for supervised fine-tuning or RL objectives that target correctness, efficiency and robustness.

### 8.4. Improving the feedback signal and granularity

Currently, IPP relies on aggregate performance metrics and a small set of worst-performing instances to guide refinement. Future work could explore more fine-grained feedback signals, such as automatically identifying which components of the synthesized program are responsible for failures or annotating traces with localized error diagnostics. There is also an opportunity to incorporate expert human feedback, which could significantly enhance IPP's performance on tasks that are difficult to verify or too complex to reduce to a single numerical metric. Such signals could make refinement more targeted and reduce the number of iterations required to reach high performance. Concretely, the coarse task-level signal could be replaced with programmatically generated rich feedback, for example, an execution trace that pairs each action with its outcome or snapshots of the grid state at the points where important actions or errors occur. Such feedback requires no additional supervision, since it can be produced automatically by instrumenting the environment. A complementary direction is to introduce a critic LLM, similar to the one used in RL-GPT [52], which could take the previous code together with such an execution trace and return more guided feedback in natural language. Recent work suggests that using an LLM as a judge can be a viable source of feedback in well-defined settings such as ours [53,54], although its reliability in this planning context would need to be established empirically.

## 9. Related work

### 9.1. Large language models for code generation and planning

LLMs have demonstrated significant capability in code generation and other code-related tasks, such as bug fixing and program testing [22,55–59]. This makes LLMs attractive for planning tasks, where structured reasoning, abstraction and correct execution are critical. Recent frameworks use LLMs to generate and refine plans by combining high-level reasoning with feedback from control or execution [60–62]. It has been shown that LLMs can translate natural language instructions into planning formalisms like Planning Domain Definition Language [63,64], or synthesize generalized planners that solve novel tasks via CoT reasoning and iterative debugging [65]. In embodied AI, LLMs have been used to generate code that serves as executable policies for robot control [28,66]. RL-GPT [52] is a framework aimed at planning and achieving a goal that requires online LLMs (fast and slow agents) to take actions, observe the effects in the environment and take further actions. The fast agent continues to refine the code based on the feedback from a critic model to complete a certain subtask. More specifically, the code specifies a list of actions (‘Python-coded subactions’) where each snippet addresses a small slice of the task, prompting repeated LLM calls. This progress suggests the feasibility of using LLMs to produce interpretable and adaptive policies for real-world tasks.

Several recent systems further demonstrate the effectiveness of treating code as a reusable policy rather than a one-off plan. Voyager [33] introduces an open-ended embodied agent that continuously generates, executes and refines Python code skills that function as policies, storing successful behaviours in a growing skill library for lifelong learning and transfer across tasks. Similarly, GenSwarm [67] proposes an end-to-end framework in which LLMs generate interpretable, white-box code policies for multi-robot teams, enabling zero-shot generalization and scalable deployment without task-specific retraining. These works highlight the growing trend of using executable code as the primary interface between language models and environments.

Other approaches emphasize neurosymbolic structure rather than direct policy generation. WorldCoder [34], for example, uses an LLM to iteratively write and update a code-based world model that captures environment dynamics, separating model learning from planning and enabling efficient transfer across grid-world tasks. While effective, such methods focus on environment modelling rather than direct synthesis of reusable control policies.

However, much of the prior work has focused on generating action plans for specific instances, often assuming strong task priors. These methods typically emphasize plan generation over policy synthesis and often lack mechanisms for generalization across varied problem settings. By contrast, our approach treats code as a reusable policy that can adapt to multiple instances within a task. We move beyond static plan generation and instead use LLMs to produce and improve executable programs based on observed performance. Importantly, our method requires an LLM only during the initial code generation and refinement phase. Once the code is generated, it can be reused any number of times and the LLM is no longer needed.

### 9.2. Iterative code generation

Recent work on iterative code generation LLMs has shown significant progress in code quality and reliability through repeated cycles of feedback and correction [68,69]. More specifically, the SELF-REFINE framework showed that LLMs can iteratively improve their outputs by generating feedback and refining their own code, achieving up to 13% improvement [70]. Building on this idea, later approaches automate refinement using external signals such as execution feedback from test suites [71], compiler diagnostics [72] and preference-guided comparisons of successful versus failed generations [73]. Additionally, some research explores multi-agent and IR, where LLMs act as solvers, verifiers or critics. This includes self-play for code and test generation [74,75], critique-driven updates [76] and counterexample-guided refinement in inductive synthesis [77].

IR has also been explored beyond traditional coding tasks. Reflexion [78] introduces a self-reflective loop in which LLM agents generate natural-language critiques of their own failures and use these reflections to improve future behaviour, yielding strong gains in sequential decision-making and code generation without parameter updates. Program-of-thoughts [79] prompting similarly treats generated code as an explicit reasoning and execution medium, separating high-level reasoning from computation and demonstrating improved reliability on complex reasoning tasks.

While prior work applies iterative methods to typical coding tasks like function completion or bug fixing, our approach extends this paradigm to planning. Instead of refining code for correctness, we use task-level metrics like energy or reward from failures to iteratively guide program generation. Our method improves entire policy programs that can handle a range of environments, not just small code pieces. Unlike test-suite-driven refinement, our framework treats code as a control policy and refines it based on real task performance in an environment. This positions our work at the intersection of LLM-based planning, code-as-policy representations and performance-driven IR.

## 10. Conclusion

In this paper, we introduced *IPP*, a framework that reconceptualizes planning in grid-based environments as code generation. By synthesizing executable Python programs rather than raw action sequences, IPP draws on the strengths of LLMs in symbolic reasoning while addressing their weaknesses in state tracking and long horizon planning. The addition of IR enables LLMs to incrementally improve generated policies through feedback, producing solutions that are interpretable, reusable and cost efficient.

Our experiments on challenging benchmarks, such as GRASP and MiniGrid, show that IPP consistently outperforms DG and non-code prompting baselines, achieving substantial improvements in both task success rates and policy transparency. Furthermore, our structured prompting strategies (pseudocode extension and curriculum-based synthesis) demonstrate how guided code generation can unlock significant performance gains across multiple LLMs.

Taken together, these results suggest that code-driven approaches offer a practical and scalable alternative to RL in symbolic domains. By grounding high-level reasoning into executable programs, IPP provides a pathway towards building planning agents that are more transparent, generalizable and efficient.

## Data Availability

Data and relevant code for this research work are stored in GitHub: http://github.com/Mystic-Slice/LLM-Planning-CodeGen and have been archived within the Zenodo repository [80]. All scripts required to reproduce the results, including data processing, simulation and analysis, are included.

## Footnotes

^1^
All prompt templates for the methods described in this section are provided in appendix C(i), (ii), (iii) and (iv) (see supplementary material, appendix C).

^2^
We omit GPT-o1 in the baseline experiments owing to cost constraints.
